# Feature selection translates drug response predictors from cell lines to patients

**DOI:** 10.3389/fgene.2023.1217414

**Published:** 2023-07-14

**Authors:** Shinsheng Yuan, Yen-Chou Chen, Chi-Hsuan Tsai, Huei-Wen Chen, Grace S. Shieh

**Affiliations:** ^1^ Institute of Statistical Science, Academia Sinica, Taipei, Taiwan; ^2^ Bioinformatics Program, Taiwan International Graduate Program, Academia Sinica, Taipei, Taiwan; ^3^ College of Medicine, Graduate Institute of Toxicology, National Taiwan University, Taipei, Taiwan; ^4^ Genome and Systems Biology Degree Program, Academia Sinica and National Taiwan University, Taipei, Taiwan; ^5^ Data Science Degree Program, Academia Sinica and National Taiwan University, Taipei, Taiwan

**Keywords:** cancer, domain adaptation, drug response, feature selection, machine learning, prediction

## Abstract

Targeted therapies and chemotherapies are prevalent in cancer treatment. Identification of predictive markers to stratify cancer patients who will respond to these therapies remains challenging because patient drug response data are limited. As large amounts of drug response data have been generated by cell lines, methods to efficiently translate cell-line-trained predictors to human tumors will be useful in clinical practice. Here, we propose versatile feature selection procedures that can be combined with any classifier. For demonstration, we combined the feature selection procedures with a (linear) logit model and a (non-linear) K-nearest neighbor and trained these on cell lines to result in LogitDA and KNNDA, respectively. We show that LogitDA/KNNDA significantly outperforms existing methods, e.g., a logistic model and a deep learning method trained by thousands of genes, in prediction AUC (0.70–1.00 for seven of the ten drugs tested) and is interpretable. This may be due to the fact that sample sizes are often limited in the area of drug response prediction. We further derive a novel adjustment on the prediction cutoff for LogitDA to yield a prediction accuracy of 0.70–0.93 for seven drugs, including erlotinib and cetuximab, whose pathways relevant to anti-cancer therapies are also uncovered. These results indicate that our methods can efficiently translate cell-line-trained predictors into tumors.

## Introduction

Targeted therapies and chemotherapies are prevalent in cancer treatments. About 7% (11.1%) of US patients with advanced or metastatic cancer benefited from genome-targeted (genome-informed) therapy in 2021 (Haslam et al., 2021), with 13.6% (27.3%) estimated eligibility. Thus, identification of the characteristics of cancer patients who will respond to chemotherapies or targeted therapies using their molecular profiles is important for precision medicine. Given that patient drug response data relative to cell lines are limited, obtaining this information is challenging. However, large-scale drug sensitivity screens of cell lines have identified clinically meaningful gene–drug interactions (Barretina et al., 2012; Garnett et al., 2012; Basu et al., 2013; Seashore-Ludlow et al., 2015). In particular, the Cancer Cell Line Encyclopedia (CCLE) database consists of the transcriptomic profiles, chromosomal copy number, and mutational profiles of 947 human cancer cell lines screened with 24 targeted therapies. Moreover, Iorio et al. published valuable results and the Genomics of Drug Sensitivity in Cancer (GDSC) dataset (Iorio et al., 2016), consisting of the multi-omics profiles and drug sensitivity scores (IC_50_) of 1,001 cancer cell lines screened with 265 anti-cancer compounds, which may be used to train predictors to improve drug response prediction in patients. Geeleher et al. (2014) adopted this approach and showed that the trained ridge regression models using whole-genome gene expression and the response of ∼700 cell lines in the Cancer Genome Project (Garnett et al., 2012) resulted in equally good or better predictions of human tumors than gene signatures derived directly from three clinical datasets.

Recently, a deep neural network-based method known as multi-omics late integration (MOLI) was proposed (Sharifi-Noghabi et al., 2019). This method was used to predict drug response by first embedding each multi-omics data type separately and then concatenating all embeddings into one representation, which was optimized via a cost function. The performance of MOLI was validated on patient-derived xenograft (PDX)/human tumor datasets of five chemotherapies and two targeted therapies. Moreover, data from cell lines screened with drugs targeting the same pathway, pan-drug data, were also integrated into MOLI to significantly improve its performance on targeted therapies.

Gene expression data are the most effective of the four omic data types for pan-cancer drug response prediction (Iorio et al., 2016). Thus, we used gene expression data in this study. Furthermore, when transferring the trained predictors from cell lines (the source domain) to human tumors or PDXs (the target domain), it is assumed that the features (genes in this study), which are used to train the predictors, behave similarly in these domains. However, cell lines and human tumors/PDXs are known to be different in the following respects (Gillet et al., 2013): there is no tumor micro-environment and vasculature in cell lines and no immune system in cell lines/PDXs. Nevertheless, strong positive correlations for mutational and transcriptomic profiles were found between cell lines and tumors (Barretina et al., 2012). Thus, it is reasonable to assume that there is a subset of genes that behave similarly between cancer cell lines and primary tumors (the two domains). PRECISE, a domain adaptation-based method, was developed to capture information shared among the preclinical models and human tumors (Mourragui et al., 2019). The resulting domain-invariant predictors were shown to reliably recover known associations between biomarkers and the corresponding drugs in human tumors. PRECISE assumes that the conditional distributions for drug response are the same in both domains ( $P_{S}\left(Y|X\right)=P_{T}\left(Y|X\right)$ ), similar to other existing methods. However, when this assumption is not met, a negative transfer will occur (Peres da Silva et al., 2021), e.g., drugs that are effective *in vitro* but not effective in clinical trials/practice.

To alleviate this shortcoming, we adopted supervised domain adaptation (DA) (Koniusz et al., 2017; Motiian et al., 2017). Although DA uses information of all labels in a test set, it has been shown to outperform numerous baselines on real-world datasets in active learning, e.g., the MNIST and USPS datasets containing images of digits from 0 to 9 analyzed by Motiian et al. (2017) and the office dataset, which is a benchmark for visual domain adaptation (Motiian et al., 2017). This study is the first application of DA to the area of drug response prediction. Here, we propose feature selection procedures combined with a regular logistic ridge regression model (called LogitDA) or with a non-linear classifier K-nearest neighbor (called KNNDA), which have the following desirable properties: 1) our assumption is weaker than that of the existing methods, and if the given training and test datasets satisfy the assumption, the proposed predictors achieve a high area under the receiver operating characteristic curve (AUC; Results), even when the training dataset is relatively small; 2) we devise an adjustment of the prediction probability cutoff for LogitDA, which leads to high prediction accuracy no matter whether datasets meet the assumption or not; and 3) the proposed method can be combined with any linear or non-linear classifier to be trained, thus being versatile.

**FIGURE 1:**
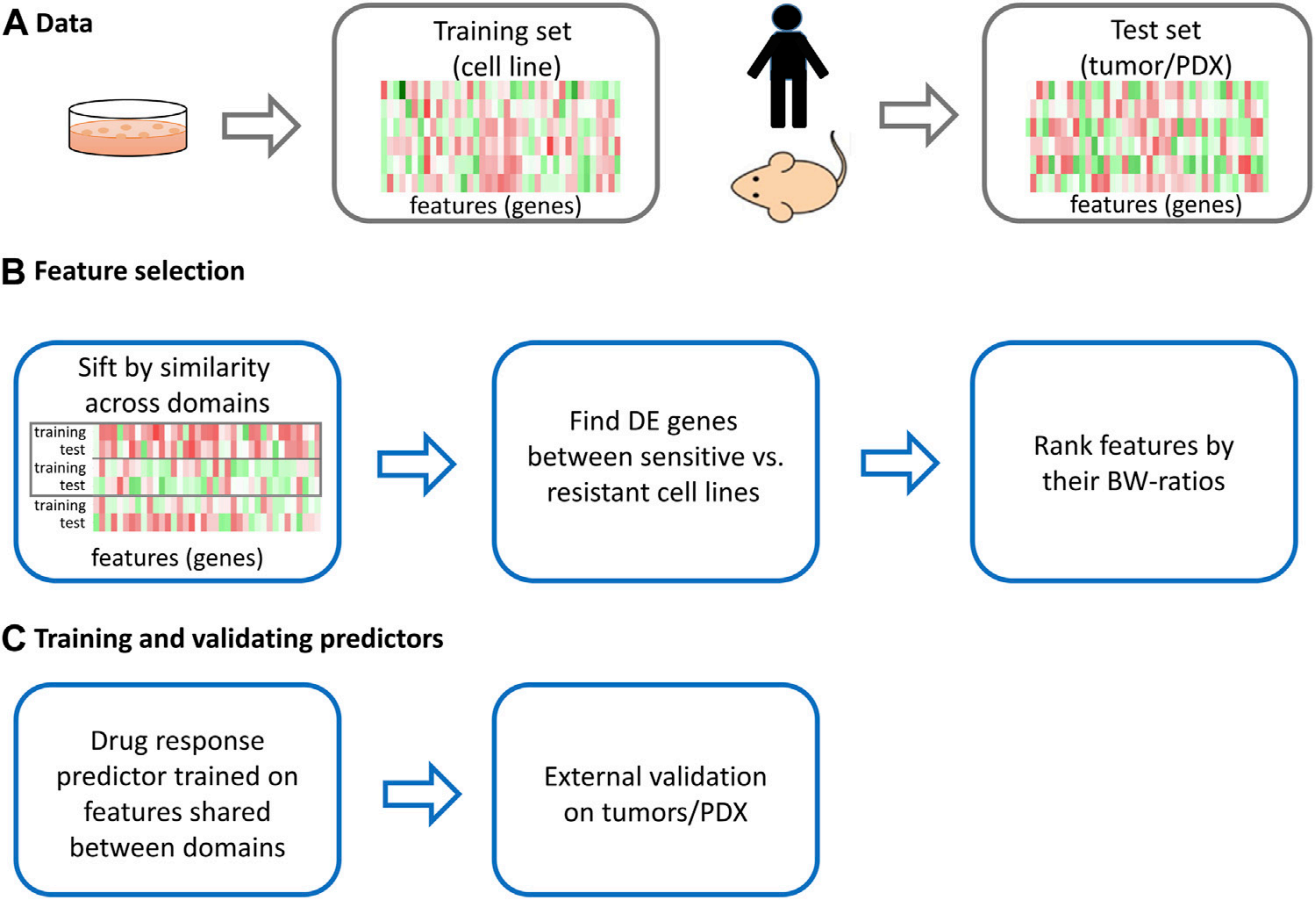
The scheme of the proposed approach.

We used the labels of the test sets only in the feature selection procedures. Specifically, we selected genes ( $X_{i}$ 's) that have similar conditional distributions across the domains, $P_{S}\left(X_{i}|Y\right)\approx P_{T}\left(X_{i}|Y\right)$ , where *S* and *T* denote the source (training) and target (test) domains, respectively. This approach falls into one category of inductive transfer learning (Pan and Yang, 2010), in which the target and source domains are labeled, but the domains and the tasks are different. In the area of drug response prediction, the source domain consists of the gene expression data of cell lines and the associated probability distribution, and its task is to predict the drug response in terms of IC_50_ scores. While the target domain consists of gene expression data of patients/PDX and the associated probability distribution, the target task is to predict the drug response of patients/PDX, which is measured by changes in tumor volumes or months-to-progress of patients. As there is no tumor micro-environment and immune system in cell lines, we assume that the probability distribution of the source and target domains is not the same. Thus, the domains and tasks are different in the area of drug response prediction. The remaining steps of our feature selection are prioritizing genes by their differential expression in sensitive *versus* resistant cell lines, keeping the top-ranked 1,000 genes for explainable features, and ranking these genes by a measure of their power to separate sensitive from resistant cell lines. Next, for each of the ten drugs, we trained a regular logit model and K-nearest neighbor (KNN) using expression data of the top-ranked *p* (*p* ≤ 1,000) genes of cell lines in GDSC via 5-fold cross-validation (CV); see Methods for details. Subsequently, we compared the performance of LogitDA/KNNDA to that of the baseline ridge regression in the work of Geeleher et al. (2014) and MOLI (Sharifi-Noghabi et al., 2019) for ten test sets in human tumors and PDXs. The scheme of the proposed approach is shown in Figure 1.

Notably, LogitDA and KNNDA turned out to be very powerful for drug response prediction. For example, LogitDA (KNNDA), trained by the top-ranked 50 (220) selected genes of the 370 cell lines screened with erlotinib, resulted in a prediction AUC of 0.94 (0.90) for NSCLC tumors. The high prediction power of LogitDA (KNNDA) for targeted therapies suggests that these predictors may help physicians stratify patients with NSCLC who will respond to erlotinib and spare those who do not from adverse effects, illustrating that these predictors have clinical implications. Finally, we uncovered the pathways of the top-fitted genes of LogitDA/KNNDA for erlotinib and cetuximab, which include pathways relevant to anti-cancer therapies and several metabolic pathways. These results indicate that our methods can efficiently translate cell-line-trained predictors into human tumors.

## Methods

### Datasets

The gene expression data and drug response of 1,001 cell lines screened with 265 drugs in the GDSC dataset (Iorio et al., 2016) were used as training sets, and ten sets of gene expression data and the response of PDX/patients treated with chemotherapies and targeted therapies were used for testing. The test sets consisted of three clinical trial datasets for docetaxel, erlotinib, and sorafenib (Geeleher et al., 2014), four sets from PDX Encyclopedia datasets (Gao et al., 2015), and three sets of TCGA patients (Weinstein et al., 2013; Ding et al., 2016). The sources for both training and testing datasets are detailed in Data Availability Statement. All datasets are publicly available.

Gene expression profiles of cell lines, which were RMA-normalized, log-transformed, and aggregated to the level of genes, were downloaded from the GDSC database. The gene expression profiles of the first three test datasets were preprocessed by Geeleher et al. (2014), and those of the remaining seven test sets (from MOLI) were converted to TPM and log-transformed by Sharifi-Noghabi et al. (2019).

### Pre-processing of gene expression data

The GED of cell lines from the GDSC dataset was first standardized by the mean and standard error (s.e.) of each gene. Next, the GED of each cell line was normalized by the house-keeping gene GAPDH across cell lines and homogenized with the GED of test sets by the ComBat() function from the sva library in R (Geeleher et al., 2014). The details of each dataset, such as the drug name, the number of samples, and the number of genes in the training and test sets, are provided in Table 1. Similar to existing methods (Geeleher et al., 2014; Sharifi-Noghabi et al., 2019), we included only genes present in both training and test sets for the subsequent analysis; the four columns from the right-hand side of Table 1 show the number of overlapping genes.

**TABLE 1:** Information about the training and test datasets of the studied drugs.

	Training set (GDSC)			Test set			No. of overlapping genes		Geeleher et al.	
Drug (test dataset)	No. of cell lines	NS^a^	NR^a^	No. of samples	NS	NR	In training and test sets^b^	Sifted by DA^c^	No. of genes in common^b^	No. of the remaining genes^d^
Docetaxel (GSE6434)	850	564	286	24	10	14	7,963	5,173	7,964	6,371
Erlotinib (GSE33072)	370	28	342	25	11	14	16,898	12,264	16,760	13,408
Sorafenib (GSE33072)	403	117	286	37	21	16	16,898	12,961	16,760	13,408
Cetuximab (PDX)	877	40	837	60	5	55	16,191	7,509	15,121	12,096
Erlotinib (PDX)	370	28	342	21	3	18	16,190	10,343	18,232	14,585
Gemcitabine (PDX)	866	680	186	25	7	18	16,190	10,115	18,232	14,585
Paclitaxel (PDX)	399	284	115	43	5	38	16,190	8,548	18,232	14,585
Cisplatin (TCGA)	850	275	575	66	60	6	16,026	8,550	18,216	14,572
Docetaxel (TCGA)	850	564	286	16	8	8	16,168	12,968	18,216	14,572
Gemcitabine (TCGA)	866	680	186	57	21	36	16,003	9,728	18,216	14,572

^a^ NS and NR denote the number of sensitive (responder) and resistant (non-responder) samples.

^b^ The number of overlapping genes between the training and test sets. The initial input genes of the work of Geeleher et al. (2014) were the same as our method, as we could not assess the input genes of the former.

^c^ The number of genes which distributed similarly, namely, filtered by supervised domain adaptation (*p* > 0.05); Kolmogorov–Smirnov test).

^d^ The number of genes remained after removing genes with the lowest 20% variability in expression across all samples.

### Feature selection procedures

For a given drug *d*, let $\left\{X_{d},Y_{d}\right\}$ consist of $X_{d}\in R^{n_{d}\times p}$ expression profiles of p genes of $n_{d}$ cell lines (PDXs or human tumors) and drug response values $Y_{d}\in \left\{0\right.,{\left.1\right\}}^{n_{d}}$ for drug *d*. Here, we dichotomized the drug response of cell lines (IC_50_) into 0 (resistant) or 1 (sensitive) if a drug response is greater or less than or equal to its maximum drug concentration (given in the GDSC website), respectively (Iorio et al., 2016).

The proposed feature selection consisted of three procedures: 1) supervised DA (Mourragui et al., 2019), 2) differential expression between sensitive and resistant cells, and 3) the ratio of “between-group to within-group sums of squares” (the BW ratio) (Dudoit et al., 2002), where the two groups refer to sensitive cell lines and resistant cell lines for each drug. As cell lines (the training sets) are different from patients and mouse models in the test sets, we applied DA to sift genes whose conditional distributions given the label *Y* across domains were not significantly different. The intuition for feature selection procedures 2) and 3) is stated in Supplementary methods. In a pilot study, we also studied Logit (KNN) trained by genes and sifted by unsupervised DA in combination with the latter two proposed feature selection procedures. However, the predictors employing unsupervised DA performed in a manner considerably inferior to those employing supervised DA, given that the remaining procedures were kept the same. Thus, we used supervised DA in this study.

Specifically, for each drug and gene *X*, the Kolmogorov–Smirnov (KS) test for equality of the conditional distribution of selected genes ***X*** given the label *Y* in the source and target domains, $F_{S}\left(X|Y\right)$ and $F_{T}\left(X|Y\right)$ , was conducted at *P* ≥α , where α=0.6,0.7,0.8 , and 0.9. It should be noted that DA used all the information about the responses of the samples in both domains. In this study, we used stringent cutoffs *P* ≥α and α=0.6,0.7 *,* 0.8, and 0.9, as the source (cell lines) and target (PDX/patients) domains are quite different. Let $X_{i}$ denote gene i. It should be noted that the aforementioned KS test is equivalent to using the distance measure $\sup\left|F_{S}\left(X_{i}|Y=1\right)-F_{T}\left(X_{i}|Y=1\right)\right|$ and $\sup\left|F_{S}\left(X_{i}|Y=0\right)-F_{T}\left(X_{i}|Y=0\right)\right|$ , where $F_{S}\left(X_{i}|Y\right)\;and\;F_{T}\left(X_{i}|Y\right)$ denote the conditional distribution of $X_{i}$ given *Y* in the source and target domains, respectively.

Given the features $X=\left(X_{1},X_{2},\ldots ,X_{p}\right)$ , we define $P_{S}\left(Y=1\right)$ and $P_{T}\left(Y=1\right)$ to be the population proportion of responders in the source and target domains, respectively. Furthermore, let ${\hat{P}}_{S}\left(Y=1|X\right)$ and ${\hat{P}}_{T}\left(Y=1|X\right)$ be the estimated proportions of responders given ***X*** in the source and target domains, respectively, and $r=\left[P_{S}\left(Y=1\right)/P_{S}\left(Y=0\right)\right]/\left[P_{T}\left(Y=1\right)/P_{T}\left(Y=0\right)\right]$ be the odds ratio of these two domains. Proposition 1 states that if the selected features satisfy the required DA condition and the odds ratio equals to 1, then the features are invariant across the source and target domains and *vice versa*.

Proposition 1 assumed that the features $X=\left(X_{1},X_{2},\ldots ,X_{p}\right)$ satisfy the DA condition and marginal conditional distributions of $X_{i}|Y$ are independent for *i =1, …, p*. Then, $P_{S}\left(Y=1|X\right)=P_{T}\left(Y=1|X\right)$ if and only if *r* = 1.

The proof is given in Supplementary information.

Genes that passed the DA selection were then prioritized by their differential expression among all overlapping genes in sensitive cell lines *versus* resistant cell lines. To reveal explainable classifiers, we kept at most the top-ranked 1,000 genes with the smallest false discovery rate (FDR) values obtained from the two-sample t-test and sorted these genes by the BW ratio. The BW ratio for a gene *j* of the cell line *i* in group *k* is defined as follows:

$$BW\left(j\right)=\frac{\underset{i}{\sum }\underset{k}{\sum }I\left(y_{i}=k\right){\left({\bar{x}}_{kj}-{\bar{x}}_{.j}\right)}^{2}}{\underset{i}{\sum }\underset{k}{\sum }I\left(y_{i}=k\right){\left({x_{ij}-\bar{x}}_{kj}\right)}^{2}},$$

where ${\bar{x}}_{.j}$ and ${\bar{x}}_{kj}$ denote the average expression level of gene *j* across all cell lines and the cell lines of group *k* only, respectively.

### Training models using GDSC datasets

For fixed top-ranked *p* genes, where *p* ranged from 50 with step size 10 to 200 (denoted by 50 (10)200), 200 (20)400, and 400 (100)1,000) genes of the cell lines, we trained the hyperparameter λ (the penalty constant of logit regression) and *p* using 5-fold CV with ten repeats. We used grid-search to tune the hyper-parameter as follows. First, let $\lambda =10^{a_{0}}$ and $a_{0}\in \left[-3,0\right]$ with step size 10^0.3^, i.e., we ran 5-fold CV of LogitDA with grid points 10^–3^, 10^–2.7^, …, and 10^0^ and found the grid point whose associated CV score was the maximum, which was termed $10^{a_{1}}$ , e.g., $a_{1}=-2.7$ . Second, we further evaluated LogitDA with grid points in $\left[10^{a_{1}-0.05},10^{a_{1}+0.05}\right]$ and step size 10^0.01^. The grid point $10^{a_{\max}}$ , whose corresponding CV score is the maximum, determines the tuned hyperparameter $\lambda =10^{a_{\max}}$ . The logit model with the highest averaged CV AUC determined *p* and λ, which yielded one LogitDA. Then, we applied the LogitDA to a test set to determine its prediction AUC. The aforementioned procedures were repeated ten times (with different seeds for CV) to obtain the mean and s.e. of the prediction AUC.

For the classifier KNN, we used the distance measure 1 − rho, where rho is Spearman’s rho between any two cell lines with selected *p* genes because the default Euclidean distance did not work well for the GED of any two cell lines in our pilot study. For each drug and fixed top-ranked *p* gene, where *p* = 50 (10)200, 200 (20) 400, and 400 (100)1,000 genes of the cell lines, we first trained the hyperparameter K of KNN, via 5-fold CV with ten repeats and using the GED and drug response of cell lines, and computed the AUC in the cross-validation experiments. The hyperparameter K was determined by the experiment with the highest averaged CV score. We then fitted all data into this KNN classifier with each top-ranked *p* gene. Of all the top-*p*-ranked KNN classifiers trained, the one with the highest averaged CV score determined the value of *p,* which was one trained KNNDA predictor. We repeated the aforementioned procedures ten times to yield the mean and s.e. of the prediction AUC of KNNDA.

### Adjustment of the probability cutoff

The following lemma and Proposition 2 established the theoretical foundation for adjusting the prediction probability cutoff when the drug response rates between cell lines and tumors differ.

Lemma. When r>1 and $P_{S}\left(Y=1|X\right)>P_{T}\left(Y=1|X\right)$ , the prediction probability is overestimated. Similarly, when r<1 and $P_{S}\left(Y=1|X\right)<P_{T}\left(Y=1|X\right)$ , the prediction probability is underestimated.

The proof is given in Supplementary information.

Proposition 2 assumed that the predictors $X=\left(X_{1},X_{2},\ldots ,X_{p}\right)$ satisfy the DA condition and marginal conditional distributions of $X_{i}|Y$ are independent for *i =1, …, p*. When the odds ratio between the source and target domains is r≠1 , the cutoff of the prediction probability $P_{S}\left(Y=1|X_{T}\right)$ should be adjusted to $r/\left(r+1\right)$ .

The proof is given in Supplementary information.

As Proposition 2 suggests, the prediction probability cutoff will deviate from 0.5 when the population proportion of responders in the target and source domains differ. Due to a lack of information on the ratio of responders in the target domain (patients), we estimated it using test data directly in this study. However, a better estimate of the ratio can be obtained once more responses to these drugs are released. We note that Proposition 2 considers a continuous prediction probability function. It does not apply to the KNN classifier that makes a prediction on a test sample based on the majority voting for its K-nearest neighbors.

### External validation of the trained classifiers

Finally, we applied the trained predictors LogitDA with *α =* 0.7 and KNNDA with *α =* 0.7 to the ten external test sets. To compare with the results of the baseline ridge regression (Geeleher et al., 2014) and MOLI complete (Sharifi-Noghabi et al., 2019), we repeated the experiments ten times for estimating the s.e. of the predictors for each drug.

## Results

### Experimental design

In this study, we aimed to investigate the following questions: Do logistic ridge regression and KNN with adequately selected features outperform a deep learning-based predictor, MOLI complete (Sharifi-Noghabi et al., 2019), in terms of prediction AUC on external test sets (PDX and patient data)? Do the proposed predictors, LogitDA and KNNDA, work well for targeted therapies and/or chemotherapies? Information about the training and test datasets of the ten studied drugs is provided in Table 1.

After the features (namely, genes in this study) were selected by the proposed procedures (see Methods for details), we trained logistic ridge regression (KNN) with 5-fold CV using the GED of the prioritized features of GDSC cell lines screened with seven drugs, which included docetaxel, erlotinib, sorafenib, cetuximab, gemcitabine, paclitaxel, and cisplatin, in a total of ten sets. These drugs were chosen because we planned to compare LogitDA and KNNDA to the baseline logistic ridge regression (Geeleher et al., 2014) and MOLI complete (Sharifi-Noghabi et al., 2019).

### Training our predictors LogitDA and KNNDA

As the training set (GDSC cell lines) is quite different from the test sets (PDX and patient data) (Sharifi-Noghabi et al., 2019), the cutoff for domain adaptation should be strict. Nevertheless, this threshold should allow sufficient features to pass so that a classifier can be adequately trained; see Methods for details. Therefore, we trained LogitDA with features ( $X_{i}$ ) that passed the KS test for equality of $F_{S}\left(X_{i}|Y\right)$ and $F_{T}\left(X_{i}|Y\right)$ with *P* ≥ α , where *α* = 0.6, 0.7, 0.8, and 0.9, and we denote the resulting predictor as LogitDA__α_, where α *=* 0.6*,* 0.7*,*0.8*,* and 0.9.

Supplementary Table S1 shows that the CV scores of LogitDA__0.6_ were equivalent to those of LogitDA__0.7_ for the ten drugs. However, to satisfy the DA condition required by Proposition 1, namely, the marginal conditional distribution of selected genes ***X*** given the label *Y* in both domains is equal, the value of *α* should be large, so we chose LogitDA__0.7_. Proposition 1 shows that features that satisfy the conditions will be domain-invariant. That is, if the features perform well in the source domain, they will also perform well in the target domain.

Moreover, we observed that each averaged CV score of LogitDA__0.7_ was higher than that of LogitDA__0.8_ and LogitDA__0.9_, except that they had the same CV score for cetuximab (PDX). Thus, among the LogitDA predictors, we suggest using LogitDA__0.7_ for the prediction of the drug response of patients/PDX and denote it by LogitDA, henceforth, for simplicity; for details, we refer to Table 2.

**TABLE 2:** Cross-validation result of LogitDA__α_ with various cutoffs of the KS test.

Method	LogitDA__0.70_			LogitDA__0.80_			LogitDA__0.90_		
	*p* ^a^	λ	CV score	*p*	λ	CV score	*p*	λ	CV score
Drug (test set)	Genes^b^			Genes			Genes		
Docetaxel (GSE6434) n = 24	170	0.447	0.76	170	0.242	0.75	50	0.424	0.660
	437			223			59		
Erlotinib (GSE30072) n = 25	50	1.039	0.80	100	0.962	0.78	50	1.122	0.677
	860			391			90		
Sorafenib (GSE30072) n = 37	110	1.122	0.68	90	1.122	0.67	50	1.122	0.633
	1,000			752			207		
Cetuximab (PDX) n = 60	130	0.019	0.86	150	0.041	0.84	50	0.048	0.802
	827			440			157		
Erlotinib (PDX) n = 21	50	0.495	0.85	50	0.521	0.82	50	1.122	0.786
	877			499			163		
Gemcitabine (PDX) n = 25	100	0.414	0.71	70	0.521	0.69	50	0.242	0.666
	1,000			541			151		
Paclitaxel (PDX) n = 43	50	0.221	0.69	50	1.122	0.64	50	1.039	0.493
	643			311			78		
Cisplatin (TCGA) n = 66	130	0.224	0.71	110	0.192	0.67	50	1.066	0.598
	628			293			79		
Docetaxel (TCGA) n = 16	650	0.521	0.79	240	0.424	0.77	80	0.236	0.741
	1,000			691			193		
Gemcitabine (TCGA) n = 57	140	0.447	0.74	70	0.192	0.72	50	0.414	0.659
	841			399			94		

^a^ *p* denotes the top-*p* genes sifted by the feature selection procedures.

^b^ Genes denote the number of genes that passed DA screening across the training and test domains for each drug.

Taking the training result of LigitDA into account, we trained the non-linear KNNDA with *α* = 0.7, 0.8, and 0.9 for the KS test and summarized the 5-fold CV result in Table 3. KNNDA__0.7_ performed better than KNNDA__0.8_, as the former had higher (lower) averaged CV scores for five (two) drugs than the latter; the differences ranged from 1% to 4%. Moreover, KNND__0.8_ outperformed KNNDA__0.9_ in terms of higher averaged CV scores for nine of the ten drugs. Thus, we suggest using KNNDA__0.7_ among these non-linear predictors for the test sets. For simplicity, we denote KNNDA__0.7_ by KNNDA henceforth.

**TABLE 3:** CV result of KNNDA__α_ with various cutoffs of the KS test.

Method	KNNDA__0.70_			KNNDA__0.80_			KNNDA__0.90_		
	*p* ^a^	Best K	CV score	*p*	Best K	CV score	*p*	Best K	CV score
Drug	Genes^b^			Genes			Genes		
Docetaxel (GSE6434) n = 24	110	23	0.76	90	21	0.74	59	19	0.69
	437			223			59		
Erlotinib (GSE30072) n = 25	220	9	0.80	140	15	0.78	50	15	0.72
	860			391			90		
Sorafenib (GSE30072) n = 37	120	17	0.64	80	19	0.65	150	19	0.60
	1,000			752			207		
Cetuximab (PDX) n = 60	110	23	0.80	50	29	0.76	50	29	0.76
	827			440			157		
Erlotinib (PDX) n = 21	60	19	0.83	200	19	0.83	110	17	0.81
	877			499			163		
Gemcitabine (PDX) n = 25	240	17	0.69	190	27	0.68	100	29	0.66
	1,000			541			151		
Paclitaxel (PDX) n = 43	100	9	0.65	80	9	0.63	60	7	0.57
	643			311			78		
Cisplatin (TCGA) n = 66	190	29	0.66	100	25	0.66	79	19	0.59
	628			293			79		
Docetaxel (TCGA) n = 16	200	25	0.75	90	19	0.76	60	29	0.74
	1,000			691			193		
Gemcitabine (TCGA) n = 57	180	29	0.67	70	29	0.67	50	27	0.63
	841			399			94		

^a^ *p* denotes the top-*p* genes sifted by the feature selection procedures.

^b^ Genes denotes the number of genes that passed DA screening across the training and test domains for each drug.

### LogitDA and KNNDA predict well for the ten drugs

**FIGURE 2:**
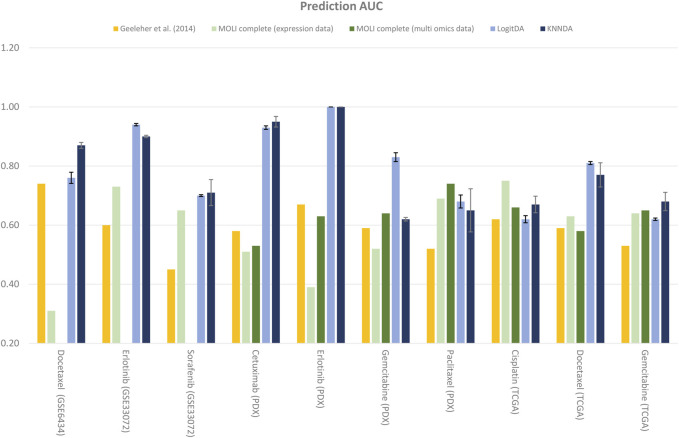
Performance of LogitDA and KNNDA compared to other methods in terms of the prediction AUC for the ten drugs.

Next, Table 4 and Figure 2 report the prediction AUC of LogitDA and KNNDA for the ten test sets. The predictor LogitDA achieved a prediction AUC >0.8 for five drugs and predicted AUCs of 0.71 and 0.70 for docetaxel and sorafenib, respectively. In particular, LogitDA using the top-ranked 50, 130, 50, 100, and 650 genes resulted in prediction AUCs of 0.94, 0.93, 1.00, 0.83, and 0.81 for erlotinib, cetuximab (PDX), erlotinib (PDX), gemcitabine (PDX), and docetaxel (TCGA), respectively. This result shows that LogitDA may be useful for precision oncology, especially for the targeted therapies erlotinib and cetuximab.

**TABLE 4:** Performance of LogitDA and KNNDA compared to the other methods in terms of prediction AUC across four targeted therapies and six chemotherapies.

Method	Geeleher et al. (2014)	MOLI complete (expression data)	MOLI complete (multi omics data)	LogitDA	KNNDA
Drug (test dataset)					
Docetaxel (GSE6434)	0.74^a^	0.31^a^	X	0.76 ± 0.019	**0.87** ^b^ ± 0.010
Erlotinib (GSE33072)	0.60	0.73	X	**0.94** ± 0.004	0.90 ± 0.004
Sorafenib (GSE33072)	0.45	0.65	X	**0.70** ± 0.003	**0.71** ± 0.044
Cetuximab (PDX)	0.58	0.51	0.53	0.93 ± 0.006	**0.95** ± 0.018
Erlotinib (PDX)	0.67	0.39	0.63	**1.00** ± 0.000	**1.00** ± 0.000
Gemcitabine (PDX)	0.59	0.52	0.64	**0.83** ± 0.015	0.62 ± 0.006
Paclitaxel (PDX)	0.52	0.69	**0.74**	0.68 ± 0.022	0.65 ± 0.073
Cisplatin (TCGA)	0.62	**0.75**	0.66	0.62 ± 0.012	0.67 ± 0.028
Docetaxel (TCGA)	0.59	0.63	0.58	**0.81** ± 0.005	0.77 ± 0.041
Gemcitabine (TCGA)	0.53	0.64	0.65	0.62 ± 0.004	**0.68** ± 0.031

^a^ The initial input genes of the work of Geeleher et al. (2014) and MOLI complete were the same as those of LogitDA and KNNDA, as we could not assess the input genes of the work of Geeleher et al. (2014). The parameters of MOLI complete were optimized using the training data.

^b^ The bold-faced values indicate the highest prediction AUC among the five methods for a drug.

Of the ten drugs, the predictor KNNDA achieved a prediction AUC >0.8 for four drugs. Specifically, KNNDA using the top-ranked 110, 220, 110, and 60 genes resulted in prediction AUCs of 0.87, 0.90, 0.95, and 1.00 for docetaxel, erlotinib, cetuximab (PDX), and erlotinib (PDX), respectively. This result shows that KNNDA may also be useful for precision oncology.

We further compared LogitDA to KNNDA. Of the ten drugs, LogitDA had a significantly higher (21% higher) (11% lower) prediction AUC compared to KNNDA for gemcitabine (PDX) (docetaxel) and performed equivalent to KNNDA for the remaining eight drugs. Thus, these predictors performed equivalently; we refer to Table 4 for details.

### Our predictors outperform the deep learning-based MOLI

As shown in Table 4 and Figure 2, the predictors LogitDA and KNNDA performed significantly better (16%–35% and 13%–37% higher prediction AUC) than the baseline logistic ridge regression model (Geeleher et al., 2014) for nine and ten out of the ten drugs, respectively.

Next, we compared LogitDA and KNNDA to the deep neural network (DNN)-based method MOLI (Sharifi-Noghabi et al., 2019), which outperformed DNNs with early integration, with 5-fold CV and 10 repeats. Of the ten drugs in Table 4, LogitDA and KNNDA outperformed MOLI complete (expression data) for seven and eight drugs, respectively. In particular, LogitDA and KNNDA had 31%–61% and 44%–61% higher prediction AUCs compared to MOLI complete for docetaxel, cetuximab (PDX), erlotinib (PDX), and gemcitabine (PDX). Furthermore, LogitDA and KNNDA also had significantly higher (18%–21% and 14%–17%) prediction AUCs compared to MOLI complete for erlotinib and docetaxel (TCGA). LogitDA and KNNDA only performed significantly worse than MOLI complete (13% and 8% lower prediction AUC) for cisplatin (TCGA).

The prediction AUC of LogitDA for both cisplatin and gemcitabine (TCGA) was only 62%, which may be because the ratio of sensitive (responders) *versus* resistant (non-responders) samples is reversed from the training to the test sets (from about 1:2 to 10:1 for cisplatin); in other words, our assumption that the ratio of sensitive to resistant samples in both domains is equal was not met.

EGFR expression has been used as a biomarker to treat colorectal cancer (CRC) patients with wild-type KRAS in the US (patients with metastatic CRC and HNSCC in the EU). However, EGFR expression does not predict a response to cetuximab (Messersmith and Ahnen, 2008). The high prediction AUC of LogitDA for cetuximab (PDX) suggests that the fitted 130 genes may be promising for selecting KRAS wild-type patients with CRC for cetuximab, provided more test sets are validated.

Furthermore, we compared LogitDA (KNNDA) to MOLI complete (multi-omics data). LogitDA (KNNDA) has a significantly higher prediction AUC 19%–40% (19%–42%) in the test set of cetuximab (PDX), erlotinib (PDX), gemcitabine (PDX, LogitDA only), and docetaxel (TCGA) and performed equivalent (<6% differences of test AUC) to MOLI complete (multi-omics data) for the remaining drugs, except that KNNDA had 9% less test AUC for paclitaxel; please refer to Table 4 for details.

For targeted therapies such as erlotinib and cetuximab, Sharifi-Noghabi and colleagues further trained MOLI on multi-omics data of five drugs targeting the EGFR pathway (MOLI complete (pan-drug)), which consisted of >3,000 samples. It is interesting that LogitDA (KNNDA) (using merely hundreds of samples) outperformed MOLI (pan-drug) for erlotinib (PDX) and cetuximab (PDX) with 28% and 13% (28% and 15%) higher prediction AUCs, respectively.

In addition to the high prediction AUC for the aforementioned drugs, our approach also has the advantages of being interpretable and using much fewer (50–650) genes that are interpretable in comparison with the baseline logistic ridge regression and MOLI, which used more than 12,000 genes, except for docetaxel, for which ∼6,370 were used, as shown in Table 1. The use of much fewer genes (features) and hyperparameters may prevent LogitDA and KNNDA from overfitting problems.

### Prediction accuracy of LogitDA for the ten drugs

For some of the ten drugs whose odds ratios of the source and target domains $r=\left[P_{S}\left(Y=1\right)/P_{S}\left(Y=0\right)\right]/\left[P_{T}\left(Y=1\right)/P_{T}\left(Y=0\right)\right]$ deviate much from 1 (Supplementary Table S2A), Proposition 2 shows that their cutoff of the predicted probability should be adjusted to r/(r+1) to account for the differences of the ratios across domains. Therefore, we adjusted the cutoffs accordingly and obtained the prediction accuracy of the ten drugs in Table 5. Notably, for seven of the ten drugs, the resulting prediction accuracy is greater than or equal to 0.70. In particular, for 25 tumors treated with erlotinib, LogitDA achieved a prediction accuracy of 0.76, and its prediction accuracy increased to 0.85 if we focused on the 20 *EGFR* and *KRAS* wild-type patients with NSCLC; LogitDA correctly predicted all 12 resistant tumors and five of eight tumors sensitive to erlotinib(Supplementary Table S2B). To the best of our knowledge, to date, there is no efficient biomarker to predict the response to erlotinib of such patients (Geeleher et al., 2014), who were estimated to represent ∼30% of Caucasian patients with lung adenocarcinoma (Wang M. et al., 2021).

**TABLE 5:** Prediction accuracy of LogitDA with the adjusted cutoffs of the ten drugs.

Drug (resource)	n^a^	Cutoff	Prediction accuracy	False positive rate	False negative rate
Docetaxel (GSE6434)	24	0.73	0.62	0.36	0.40 (4/10)
Erlotinib (GSE33072)	25	0.09	0.76	0.00	0.55 (6/11)
Sorafenib (GSE33072)	37	0.24	0.62	0.63^b^	0.19 (4/21)
Cetuximab (PDX)	60	0.34	0.93	0.00	0.80 (4/5)
Erlotinib (PDX)	21	0.33	0.86	0.00	1.00 (3/3)
Gemcitabine (PDX)	25	0.90	0.72	0.06	0.86 (6/7)
Paclitaxel (PDX)	43	0.95	0.88	0.00	1.00 (5/5)
Cisplatin (TCGA)	66	0.05	0.88	0.83^b^	0.05 (3/60)
Docetaxel (TCGA)	16	0.66	0.69	0.38	0.25 (2/8)
Gemcitabine (TCGA)	57	0.86	0.70	0.17	0.52 (11/21)

^a^ n denotes the sample size.

^b^ The false positive rate of sorafenib (GSE33072) and cisplatin (TCGA) are 10/16 and 5/6, respectively.

### Ablation study

In a pilot study, we found that DA considerably improved the prediction AUC of logistic ridge regression (KNN) combined with feature selection using DE genes and BW ratio. Thus, it was of interest to evaluate the contribution of DA. We trained logistic ridge regression and KNN with the features selected by the two aforementioned feature selections (denoted as LogitDA-DA and KNNDA-DA, respectively) for the ten drugs. Supplementary Table S3 shows that LogitDA-DA (KNNDA-DA) used a few hundred genes to achieve equivalent test AUCs as the baseline logit model trained by more than 5,100–13,400 features for the ten drugs (Figure 2). Table 6 shows that DA increases the averaged prediction AUC of LogitDA (KNNDA) from 0.55 to 0.79 (0.57–0.78) over LogitDA-DA (KNNDA-DA), where the averaged prediction AUC was averaged over the ten drugs; the improvements are quite significant.

**TABLE 6:** Ablation study of the proposed predictors with DA *versus* without DA.

Experimental setting	Averaged prediction AUC (s.e.)^a^
LogitDA	0.79 (0.14)
LogitDA-DA	0.55 (0.11)
KNNDA	0.78 (0.14)
KNNDA-DA	0.57 (0.14)

^a^ The averaged prediction AUC and its s.e. were computed over those of the ten drugs studied.

### Pathways relevant to erlotinib and cetuximab discovered

As LogitDA and KNNDA perform well in the prediction AUC for erlotinib and cetuximab (PDX), it is of interest to find the pathways in which the fitted genes of these predictors are involved. Thus, we first submitted the top-ranked 220 genes of LogitDA and KNNDA for erlotinib into the database Ingenuity Pathway Analysis (IPA; http://www.ingenuity.com) and uncovered the relevant pathways in Supplementary Table S4. Interestingly, several important metabolic pathways were discovered, e.g., Purine Nucleotides *de novo* Biosynthesis (Ali et al., 2020; Taha-Mehlitz et al., 2021) and Histidine Degradation VI (Tominaga et al., 2019; Han et al., 2020). Furthermore, pathways for epigenetic regulation (Yu et al., 2018; Du et al., 2019) and DNA repair (nucleotide excision repair enhanced pathway) (Dong et al., 2019; Wang T. et al., 2021; Sato et al., 2021) were also uncovered. The aforementioned pathways play essential roles in tumor malignancy and response to anti-cancer therapies.

The overlap of the aforementioned fitted genes and the uncovered pathways (in the molecules column of Supplementary Table S5) has been reported to contribute to tumor progression (cell proliferation, survival, invasion, and metastasis) and drug resistance. Specifically, LIG1 is an attractive target for personalization of ovarian cancer therapy (Ali et al., 2021), and decreased eEF2 phosphorylation, mediated by increased PP2A activity, contributes to resistance to HER2 inhibition (McDermott et al., 2014). ADSL has been suggested as a predictive biomarker of response to 6-mercaptopurine (under the brand name Purinethol) in a pre-clinical setting (Taha-Mehlitz et al., 2021).

Moreover, MTA3 downregulates SOX2OT, and the MTA3/SOX2-OT/SOX2 axis has been reported as a potential cancer stratification marker in human esophageal squamous cell carcinomas (Du et al., 2019). Finally, CAD, a key enzyme of *de novo* pyrimidine biosynthesis essential for cell proliferation, has been found to directly interact with the second generation of EGFR-TKI Afatinib, which also targets EGFR in the same pathway as erlotinib (Tu et al., 2021).

Similarly, we submitted the top-ranked 130 genes of LogitDA for cetuximab (PDX) and uncovered DNA repair, metabolic processes, and lysosome-associated pathways; the details of the pathways are listed in Supplementary Table S5. The overlap of the fitted genes and the uncovered pathways includes CDK7, IGF-1R, and others. In particular, CDK7 is a key regulator of transcription and cell-cycle control, and its deregulation in cancer has been linked to a worse prognosis (Jagomast et al., 2022). Inhibition of CDK7/12 promotes resistance emergence in response to targeted therapy in lung cancer cells (Rusan et al., 2018; Terai et al., 2018). Moreover, cetuximab therapeutically blocks EGFR, and this might concurrently induce the activation of IGF-1R, which could activate EGFR-downstream Akt signaling, thus mediating cetuximab resistance in gastric cancer cells (Li et al., 2015).

## Discussion

Our feature selection approach can be used in combination with any classifier or regression model, not restricted to the logistic ridge regression and KNN demonstrated here, to predict the response of cancer patients using gene expression data. In particular, the ablation study shows that DA increases the prediction power by ∼24% (21%) from LogitDA-DA (KNNDA-DA). Following standard practice, we have chosen the K value of KNNDA that yielded the largest average AUC from 5-fold CV. To see the impact of the selection of K, we computed the test AUC of KNNDA with various values of K in Supplementary Table S6 (p. 17, Supplementary File). The result shows that excluding the smallest value of K (3), within the neighborhood of the optimized K (say, K ± 3), the yielded test AUC of KNNDA deviates from the reported AUC only within 0.05, except for sorafenib (−0.08, 0), paclitaxel (−0.06, 0.04), and docetaxel (0, 0.09). This may be due to the large s.e. of KNNDA for these drugs, 0.03, 0.07, and 0.04 in the test AUC from 10 repeats (Table 4).

LogitDA (KNNDA) performed very well on prediction for five (four) out of the ten targeted therapies and chemotherapies (AUC >0.81), i.e., erlotinib (two sets), cetuximab, gemcitabine, and docetaxel. Thus, these predictors may efficiently uncover novel biomarkers and pathways, although large test sets are warranted. In addition to the high prediction AUC for the aforementioned drugs, our approach also has the advantage of using much fewer (50–650) genes than the baseline logistic ridge regression and MOLI, which used more than 5,100 or 12,000 genes.

Notably, using the novel adjusted cutoff of prediction probability, LogitDA achieved a prediction accuracy of 0.70 or higher for seven of the ten drugs. In particular, the prediction accuracy of LogitDA increases to 0.76 from 0.56 (using the default cutoff of 0.5), using the adjusted cutoff of 0.09, as Proposition 2 suggests. Moreover, its prediction accuracy increased to 0.80 if we focused on 20 *EGFR* and *KRAS* wild-type patients with NSCLC, whereas there is no currently effective predictive marker of drug response for these patients. Thus, LogitDA may be useful for stratifying such NSCLC patients for erlotinib in clinical practice. Although the test AUCs for chemotherapies such as paclitaxel and cisplatin were only 0.68 and 0.62, respectively, their prediction accuracy achieved 0.88 and 0.88 for paclitaxel (PDX) and cisplatin (66 patients in TCGA), using the adjusted cutoffs for the prediction probability.

As LogitDA and KNNDA performed well in prediction responses to erlotinib and cetuximab, we used the fitted genes of the predictors to uncover several important metabolic pathways for these drugs, in addition to DNA repair pathways. The aforementioned pathways play essential roles in tumor malignancy and response to anti-cancer therapies.

It is interesting to point out that LogitDA performed particularly well for certain targeted therapies. LogitDA used 370 cell lines for training and achieved test AUCs of 0.94 and 1.00 for erlotinib (clinical trial and PDX). In contrast, deep learning-based methods, e.g., MOLI aggregated related samples (of drugs targeting the same EGFR pathway) to a larger training set (>3,000 cell lines) and used multi omics data to train the classifier, increased the test AUC from 0.63 to 0.72 for erlotinib (PDX). These differences may be because our approach prioritizes important features, limiting the number of parameters in the logistic ridge regression to at most 1,000 genes to fit. Nevertheless, MOLI (gene expression) performed very well in the prediction of chemotherapies, e.g., with a prediction AUC of 0.75 for cisplatin (TCGA), which outperformed LogitDA (using 130 genes) and KNNDA (using 190 genes).

This study employed GED, which has been shown to be the most predictive data type among omics data (Iorio et al., 2016), to predict the drug response of cancer patients; integrating GED and other omics data types to predict the drug response is a natural extension. We postulate that chemotherapies usually target broad biological mechanisms, so predictors for these therapies may require more genes to train to predict well. This suggests a future research direction in which biological domain knowledge (Ma et al., 2021) is incorporated to integrate samples screened with several therapeutics targeting the same tumorigenesis mechanism to improve the performance of our approach. This research direction is similar to a recent development in which adversarial inductive transfer learning (Pan and Yang, 2010) is applied to drug response prediction (AITL, Sharifi-Noghabi et al., 2020). AITL applied adversarial domain adaptation and multi-task learning to tackle discrepancies in the input and output spaces of drug response prediction. Moreover, combining the proposed feature selection method with deep learning-based methods may prove powerful for drug response, as the former has been shown to improve the prediction power of linear and nonlinear predictors for drug response. Finally, applying the concept of few-shot learning (Ma et al., 2021), namely, applying DA to only partial test samples and keeping the remaining procedures the same, may reveal the minimum number of test labels required for adequate performance of our predictors.

## Data Availability

Publicly available datasets were analyzed in this study. These data can be found at: http://geeleherlab.org/cgpPrediction; https://zenodo.org/record/4036592; PDX Encyclopedia datasets; TCGA; and Broad GDAC Firehose.

## References

[B1] Ali E. S. Sahu U. Villa E. O’Hara B. P. Gao P. Beaudet C. (2020). ERK2 phosphorylates PFAS to mediate posttranslational control of de novo purine synthesis. Mol. Cell. 78 (6), 1178–1191.e6. 10.1016/j.molcel.2020.05.001 32485148 PMC7306006

[B2] Ali R. Alabdullah M. Algethami M. Alblihy A. Miligy I. Shoqafi A. (2021). Ligase 1 is a predictor of platinum resistance and its blockade is synthetically lethal in XRCC1 deficient epithelial ovarian cancers. Theranostics 11 (17), 8350–8361. 10.7150/thno.51456 34373746 PMC8344016

[B3] Barretina J. Caponigro G. Stransky N. Venkatesan K. Margolin A. A. Kim S. (2012). The Cancer Cell Line Encyclopedia enables predictive modelling of anticancer drug sensitivity. Nature 483 (7391), 603–607. 10.1038/nature11003 22460905 PMC3320027

[B4] Basu A. Bodycombe N. E. Cheah J. H. Price E. V. Liu K. Schaefer G. I. (2013). An interactive resource to identify cancer genetic and lineage dependencies targeted by small molecules. Cell. 154 (5), 1151–1161. 10.1016/j.cell.2013.08.003 23993102 PMC3954635

[B5] Ding Z. Zu S. Gu J. (2016). Evaluating the molecule-based prediction of clinical drug responses in cancer. Bioinformatics 32 (19), 2891–2895. 10.1093/bioinformatics/btw344 27354694

[B6] Dong S. Li W. Wang L. Hu J. Song Y. Zhang B. (2019). Histone-related genes are hypermethylated in lung cancer and hypermethylated HIST1H4F could serve as a pan-cancer biomarker. Cancer Res. 79 (24), 6101–6112. 10.1158/0008-5472.CAN-19-1019 31575549

[B7] Du L. Wang L. Gan J. Yao Z. Lin W. Li J. (2019). MTA3 represses cancer stemness by targeting the SOX2OT/SOX2 Axis. Iscience 22, 353–368. 10.1016/j.isci.2019.11.009 31810000 PMC6909183

[B8] Dudoit S. Fridlyand J. Speed T. P. (2002). Comparison of discrimination methods for the classification of tumors using gene expression data. J. Am. Stat. Assoc. 97 (457), 77–87. 10.1198/016214502753479248

[B9] Gao H. Korn J. M. Ferretti S. Monahan J. E. Wang Y. Singh M. (2015). High-throughput screening using patient-derived tumor xenografts to predict clinical trial drug response. Nat. Med. 21 (11), 1318–1325. 10.1038/nm.3954 26479923

[B10] Garnett M. J. Edelman E. J. Heidorn S. J. Greenman C. D. Dastur A. Lau K. W. (2012). Systematic identification of genomic markers of drug sensitivity in cancer cells. Nature 483 (7391), 570–575. 10.1038/nature11005 22460902 PMC3349233

[B11] Geeleher P. Cox N. J. Huang R. S. (2014). Clinical drug response can be predicted using baseline gene expression levels and in vitrodrug sensitivity in cell lines. Genome Biol. 15 (3), R47–R12. 10.1186/gb-2014-15-3-r47 24580837 PMC4054092

[B12] Gillet J.-P. Varma S. Gottesman M. M. (2013). The clinical relevance of cancer cell lines. J. Natl. Cancer Inst. 105 (7), 452–458. 10.1093/jnci/djt007 23434901 PMC3691946

[B13] Han M. Wang S. Yang N. Wang X. Zhao W. Saed H. S. (2020). Therapeutic implications of altered cholesterol homeostasis mediated by loss of CYP46A1 in human glioblastoma. EMBO Mol. Med. 12 (1), e10924. 10.15252/emmm.201910924 31777202 PMC6949512

[B14] Haslam A. Kim M. Prasad V. (2021). Updated estimates of eligibility for and response to genome-targeted oncology drugs among US cancer patients, 2006-2020. Ann. Oncol. 32 (7), 926–932. 10.1016/j.annonc.2021.04.003 33862157

[B15] Iorio F. Knijnenburg T. A. Vis D. J. Bignell G. R. Menden M. P. Schubert M. (2016). A landscape of pharmacogenomic interactions in cancer. Cell. 166 (3), 740–754. 10.1016/j.cell.2016.06.017 27397505 PMC4967469

[B16] Jagomast T. Idel C. Klapper L. Kuppler P. Offermann A. Dreyer E. (2022). CDK7 predicts worse outcome in head and neck squamous-cell cancer. Cancers 14 (3), 492. 10.3390/cancers14030492 35158760 PMC8833595

[B17] Koniusz P. Tas Y. Porikli F. (2017). “Domain adaptation by mixture of alignments of second-or higher-order scatter tensors,” in Proceedings of the IEEE conference on computer vision and pattern recognition, Honolulu, HI, USA, July 2017 (IEEE), 4478–4487.

[B18] Li X. Xu L. Li H. Zhao L. Luo Y. Zhu Z. (2015). Cetuximab-induced insulin-like growth factor receptor I activation mediates cetuximab resistance in gastric cancer cells. Mol. Med. Rep. 11 (6), 4547–4554. 10.3892/mmr.2015.3245 25625229

[B19] Ma J. Fong S. H. Luo Y. Bakkenist C. J. Shen J. P. Mourragui S. (2021). Few-shot learning creates predictive models of drug response that translate from high-throughput screens to individual patients. Nat. Cancer 2 (2), 233–244. 10.1038/s43018-020-00169-2 34223192 PMC8248912

[B20] McDermott M. S. Browne B. C. Conlon N. T. O’Brien N. A. Slamon D. J. Henry M. (2014). PP2A inhibition overcomes acquired resistance to HER2 targeted therapy. Mol. cancer 13 (1), 157. 10.1186/1476-4598-13-157 24958351 PMC4230643

[B21] Messersmith W. A. Ahnen D. J. (2008).Targeting EGFR in colorectal cancer. N. Engl. J. Med. 359 (17), 1834–1836. 18946069 10.1056/NEJMe0806778

[B22] Motiian S. Piccirilli M. Adjeroh D. A. Doretto G. (2017). “Unified deep supervised domain adaptation and generalization,” in Proceedings of the IEEE international conference on computer vision (IEEE), 5715–5725.

[B23] Mourragui S. Loog M. Van De Wiel M. A. Reinders M. J. Wessels L. F. (2019). Precise: A domain adaptation approach to transfer predictors of drug response from pre-clinical models to tumors. Bioinformatics 35 (14), i510–i519. 10.1093/bioinformatics/btz372 31510654 PMC6612899

[B24] Pan S. J. Yang Q. (2010). A survey on transfer learning. IEEE Trans. Knowl. Data Eng. 22, 1345–1359. 10.1109/tkde.2009.191

[B25] Peres da Silva R. Suphavilai C. Nagarajan N. (2021). Tugda: Task uncertainty guided domain adaptation for robust generalization of cancer drug response prediction from *in vitro* to *in vivo* settings. Bioinformatics 37 (1), i76–i83. 10.1093/bioinformatics/btab299 34000002 PMC8275325

[B26] Rusan M. Li K. Li Y. Christensen C. L. Abraham B. J. Kwiatkowski N. (2018). Suppression of adaptive responses to targeted cancer therapy by transcriptional repression. Cancer Discov. 8 (1), 59–73. 10.1158/2159-8290.CD-17-0461 29054992 PMC5819998

[B27] Sato M. Liebau A. W. Liu Z. Liu L. Rabadan R. Gautier J. (2021). The UVSSA complex alleviates MYC-driven transcription stress. J. Cell. Biol. 220 (2), e201807163. 10.1083/jcb.201807163 33404608 PMC7791342

[B28] Seashore-Ludlow B. Rees M. G. Cheah J. H. Cokol M. Price E. V. Coletti M. E. (2015). Harnessing connectivity in a large-scale small-molecule sensitivity dataset. Cancer Discov. 5 (11), 1210–1223. 10.1158/2159-8290.CD-15-0235 26482930 PMC4631646

[B29] Sharifi-Noghabi H. Zolotareva O. Collins C. C. Ester M. (2019). Moli: Multi-omics late integration with deep neural networks for drug response prediction. Bioinformatics 35 (14), i501–i509. 10.1093/bioinformatics/btz318 31510700 PMC6612815

[B30] Sharifi-Noghabi H. Peng S. Zolotareva O. Collins C. C. Ester M. (2020). Aitl: Adversarial Inductive Transfer Learning with input and output space adaptation for pharmacogenomics. Bioinformatics 36 (1), i380–i388. 10.1093/bioinformatics/btaa442 32657371 PMC7355265

[B31] Taha-Mehlitz S. Bianco G. Coto-Llerena M. Kancherla V. Bantug G. R. Gallon J. (2021). Adenylosuccinate lyase is oncogenic in colorectal cancer by causing mitochondrial dysfunction and independent activation of NRF2 and mTOR-MYC-axis. Theranostics 11 (9), 4011–4029. 10.7150/thno.50051 33754045 PMC7977451

[B32] Terai H. Kitajima S. Potter D. S. Matsui Y. Quiceno L. G. Chen T. (2018). ER stress signaling promotes the survival of cancer "persister cells" tolerant to EGFR tyrosine kinase inhibitors. Cancer Res. 78 (4), 1044–1057. 10.1158/0008-5472.CAN-17-1904 29259014 PMC5815936

[B33] Tominaga K. Minato H. Murayama T. Sasahara A. Nishimura T. Kiyokawa E. (2019). Semaphorin signaling via MICAL3 induces symmetric cell division to expand breast cancer stem-like cells. Proc. Natl. Acad. Sci. 116 (2), 625–630. 10.1073/pnas.1806851116 30587593 PMC6329980

[B34] Tu H.-F. Ko C.-J. Lee C.-T. Lee C.-F. Lan S.-W. Lin H.-H. (2021). Afatinib exerts immunomodulatory effects by targeting the pyrimidine biosynthesis enzyme CAD. Cancer Res. 81 (12), 3270–3282. 10.1158/0008-5472.CAN-20-3436 33771897

[B35] Wang M. Herbst R. S. Boshoff C. (2021). Toward personalized treatment approaches for non-small-cell lung cancer. Nat. Med. 27 (8), 1345–1356. 10.1038/s41591-021-01450-2 34385702

[B36] Wang T. Chen X. Jing F. Li Z. Tan H. Luo Y. (2021). Identifying the hub genes in non-small cell lung cancer by integrated bioinformatics methods and analyzing the prognostic values. Pathology-Research Pract. 228, 153654. 10.1016/j.prp.2021.153654 34749208

[B37] Weinstein J. N. Collisson E. A. Mills G. B. Shaw K. R. Ozenberger B. A. Ellrott K. (2013). The cancer genome atlas pan-cancer analysis project. Nat. Genet. 45 (10), 1113–1120. 10.1038/ng.2764 24071849 PMC3919969

[B38] Yu N. Zhang P. Wang L. He X. Yang S. Lu H. (2018). RBBP7 is a prognostic biomarker in patients with esophageal squamous cell carcinoma. Oncol. Lett. 16 (6), 7204–7211. 10.3892/ol.2018.9543 30546458 PMC6256704

