# Supplementary material for: Feature selection translates drug response predictors from cell lines to patients
Source: Front Genet. 2023 Jul 14;14:1217414. doi: 10.3389/fgene.2023.1217414 (PMC10382684; doi:10.3389/fgene.2023.1217414)
Supplement: Supplementary file 1 [file DataSheet1.pdf]

Feature selection translates drug response predictors from cell lines to patients

Shinsheng Yuan[1,2+], Yen-Chou Chen[1+], Chi-Hsuan Tsai[1], Huei-Wen Chen[3] & Grace S. Shieh[1,2,4,5*]*

Correspondence: Grace S. Shieh: gshieh@stat.sinica.edu.tw

1 Supplementary Methods

1.1 Proofs for Propositions

Proposition 1. Assume that the features $\boldsymbol{X} = (X_1, X_2, \dots, X_p)$ satisfy the DA condition and marginal conditional distributions of $X_i|Y$'s are independent, for $i = 1, \dots, p$. Then $P_S(Y = 1|\boldsymbol{X}) = P_T(Y = 1|\boldsymbol{X})$ if and only if $r = 1$.

Proof:

Since X_i's satisfy the DA conditions, $f(X_i|Y)$'s are the same across both domains.

Namely, $f_S(X_i|Y) = f_T(X_i|Y), i = 1, \dots, p$. Because $f(X_i|Y)$'s are independent, their joint distribution is the product of its marginal distributions. Thus, $f_S(\boldsymbol{X}|Y) = \prod_{i=1}^{p} f_S(X_i|Y) = \prod_{i=1}^{p} f_T(X_i|Y) = f_T(\boldsymbol{X}|Y)$.

To prove that $P_S(Y = 1|\boldsymbol{X}) = P_T(Y = 1|\boldsymbol{X})$,

$$P_S(Y = 1|\boldsymbol{X}) = f_S(\boldsymbol{X}|Y = 1)P_S(Y = 1)/[f_S(\boldsymbol{X}|Y = 1)P_S(Y = 1) + f_S(\boldsymbol{X}|Y = 0)P_S(Y = 0)]$$

$$= f_T(\boldsymbol{X}|Y = 1)P_S(Y = 1)/[f_T(\boldsymbol{X}|Y = 1)P_S(Y = 1) + f_T(\boldsymbol{X}|Y = 0)P_S(Y = 0)]$$

$$= f_T(\boldsymbol{X}|Y = 1)/[f_T(\boldsymbol{X}|Y = 1) + f_T(\boldsymbol{X}|Y = 0)(P_S(Y = 0)/P_S(Y = 1))]$$

$$= f_T(\boldsymbol{X}|Y = 1)/[f_T(\boldsymbol{X}|Y = 1) + f_T(\boldsymbol{X}|Y = 0)(P_T(Y = 0)/P_T(Y = 1))/r]$$

So, when $r = 1$,

$$= f_T(\boldsymbol{X}|Y = 1)/[f_T(\boldsymbol{X}|Y = 1) + f_T(\boldsymbol{X}|Y = 0)(P_T(Y = 0)/P_T(Y = 1))]$$

$$= f_T(\boldsymbol{X}|Y = 1)P_T(Y = 1)/[f_T(\boldsymbol{X}|Y = 1)P_T(Y = 1) + f_T(\boldsymbol{X}|Y = 0)P_T(Y = 0)]$$

$$= P_T(Y = 1|\boldsymbol{X})$$

Lemma. When $r > 1$, $P_S(Y = 1|\boldsymbol{X}) > P_T(Y = 1|\boldsymbol{X})$, and the prediction probability is over-estimated. Similarly, when $r < 1$, $P_S(Y = 1|\boldsymbol{X}) < P_T(Y = 1|\boldsymbol{X})$ and the prediction probability is under-estimated.

Proof:

$P_S(Y=1|\boldsymbol{X})$

$= f_T(\boldsymbol{X}|Y=1)/[f_T(\boldsymbol{X}|Y=1)+f_T(\boldsymbol{X}|Y=0)(P_T(Y=0)/P_T(Y=1))/r]$

So, when $r>1$,

$P_S(Y=1|\boldsymbol{X})$

$> f_T(\boldsymbol{X}|Y=1)/[f_T(\boldsymbol{X}|Y=1)+f_T(\boldsymbol{X}|Y=0)(P_T(Y=0)/P_T(Y=1))]$

$= f_T(\boldsymbol{X}|Y=1)P_T(Y=1)/[f_T(\boldsymbol{X}|Y=1)P_T(Y=1)+f_T(\boldsymbol{X}|Y=0)P_T(Y=0)]$

$= P_T(Y=1|\boldsymbol{X})$

Proposition 2. Assume that the predictors $\boldsymbol{X}=(X_1,X_2,\dots,X_p)$ satisfy the DA condition and marginal conditional distributions of $X_i|Y$'s are independent, for $i=1, \dots, p$. When the odds ratio between the source and target domains r $\neq 1$, the cutoff of the prediction probability $P_S(Y=1|\boldsymbol{X}_T)$ should be adjusted to $r/(r+1)$.

Proof:

For simplicity, we first define the following.

$g_S(\boldsymbol{X}) = f_S(\boldsymbol{X}|Y=1)/f_S(\boldsymbol{X}|Y=0)$

$g_T(\boldsymbol{X}) = f_T(\boldsymbol{X}|Y=1)/f_T(\boldsymbol{X}|Y=0)$.

L_S: $\{\boldsymbol{X}\in \mathrm{R}^p \mid g_S(\boldsymbol{X}) = P_S(Y=0)/P_S(Y=1)\}$

L_T: $\{\boldsymbol{X}\in \mathrm{R}^p \mid g_T(\boldsymbol{X}) = P_T(Y=0)/P_T(Y=1)\}$

Let surface L_S and L_T be the boundary of the predictions in their respective domains, respectively. DA implies that $g_S(\boldsymbol{X}) = g_T(\boldsymbol{X})$. The difference between these two domains will lead to separation of the two boundaries L_S and L_T. In other words, the cutoff value in the target domain will deviate from 0.5, which is the original cutoff used in the source domain, where the boundary surface for the trained model is L_S.

Let $\boldsymbol{X}_T$ be the features on the surface L_T. Thus, the prediction probabilities estimated from $P_S(Y=1|\boldsymbol{X}_T)$ of the points $\boldsymbol{X}_T$, namely the adjusted cutoff in the target domain, equals to

$P_S(Y=1|\boldsymbol{X}_T)$

$= f_S(\boldsymbol{X}_T|Y=1)/[f_S(\boldsymbol{X}_T|Y=1)+f_S(\boldsymbol{X}_T|Y=0)(P_S(Y=0)/P_S(Y=1))]$

$= g_S(\boldsymbol{X}_T)/[g_S(\boldsymbol{X}_T)+(P_S(Y=0)/P_S(Y=1))]$

$= 1/\{1+[P_T(Y=1)/P_T(Y=0)]/[P_S(Y=1)/P_S(Y=0)]\}$

$= 1/\{1 + 1/r\} = r/(r + 1)$

1.2 Hyperparameter tuning procedure for KNN classifier

For the classifier KNN, we used the distance measure 1- rho, where rho is the Spearman's rho between any two cell lines with selected p genes, because the default Euclidean distance did not work well for GED of any two cell lines in our pilot study. For each drug and the fixed top-ranked p genes, where p = 50(10)200, 200(20) 400 and 400(100)1000 genes of the cell lines, we trained the hyperparameter K of KNN, via 5-fold CV with ten repeats and using GED and drug response of cell lines, and computed AUC in the cross-validation experiments. The hyperparameter K was determined by the experiment with the highest averaged CV-score. We then fitted all data to this KNN classifier with each top-ranked p genes. Of all top-p ranked KNN classifiers trained, the one with the highest averaged CV-score determined the value of p, which was one trained KNNDA predictor. We repeated the aforementioned procedures ten times to yield the mean and s.e. of prediction AUC of KNNDA.

1.3 The intuition for feature selection procedure

To set priorities of more than 17,000 genes in the cell lines, we identified differentially expressed (DE) genes of sensitive cell lines versus resistant cell lines to a given drug, by two-sample t-tests. For example, we identified the DE genes of the 28 sensitive versus 342 resistant cell lines screened with Erlotinib, and sorted their associated p-values. The biological meaning of these differentially expressed genes lies in that these are statistically significantly associated with sensitive but not resistant to Erlotinib.

We next clustered DE genes, whose false discovery rates are < 0.05, of the 370 cell lines treated with Erlotinib. Specifically, we applied hierarchical clustering to the 447 genes with FDR < 0.05 using Spearman's rank correlation, which resulted in the following Figure S1.

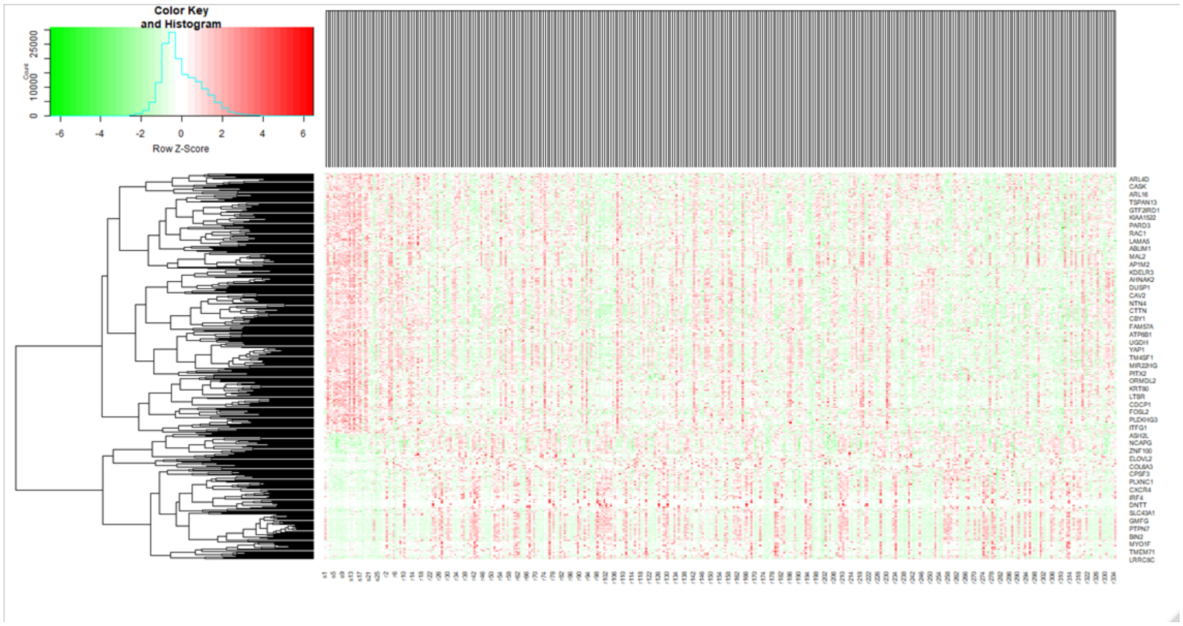

Figure S1. The clustered gene expression graph of 27 sensitive and 334 resistant cell lines screened with Erlotinib, where columns are cell lines and rows are genes, and red and green color denote over- and under-expression of genes, respectively.

The most left 27 columns of Figure S1 are cell lines sensitive to Erlotinib in GDSC, while the remaining ones are resistant to Erlotinib. This graph provided the intuition to further screen these DE genes. Namely, the genes overexpressed (under-expressed) in sensitive cells but under-expressed (overexpressed) in resistant ones were able to distinguish sensitive cells from the resistant ones. Therefore, we further ranked the DE genes by descending orders of the BW-ratios (Dudoit, Fridlyand and Speed, 2002).

2 Supplementary Figures and Tables

Table S1. The cross-validation (CV) result of LogitDA with cutoffs of the KS-test ranging from 0.6 to 0.9

Method / Drug (test set)	**LogitDA_0.6**			**LogitDA_0.7**		
	p[a] / genes[b]	λ	CV score	*p* / genes	λ	CV score (s.e.)
Docetaxel (GSE6434) n=24	200	0.508	**0.76**	170	0.447	**0.76** (0.01)
	796			437		
Erlotinib (GSE30072) n=25	70	1.039	**0.80**	50	1.039	**0.80** (0.01)
	1000			860		
Sorafenib (GSE30072) n=37	50	1.122	**0.68**	110	1.122	**0.68** (0.01)
	1000			1000		
Cetuximab (PDX) n=60	220	0.042	**0.87**	130	0.019	**0.86** (0.01)
	1000			827		
Erlotinib (PDX) n=21	50	0.101	**0.87**	50	0.495	0.85 (0.01)
	1000			877		
Gemcitabine (PDX) n=25	170	0.508	**0.72**	100	0.414	0.71 (0.003)
	1000			1000		
Paclitaxel (PDX) n=43	70	0.508	**0.72**	50	0.221	0.69 (0.01)
	1000			643		
Cisplatin (TCGA) n=66	60	0.435	**0.72**	130	0.224	0.71 (0.01)
	1000			628		
	360	0.482	**0.79**	650	0.521	0.79

Docetaxel (TCGA) n=16	1000			1000		(0.01)
Gemcitabine (TCGA) n=57	80	1.094	**0.74**	140	0.447	**0.74** (0.004)
	1000			841		

[a]*p* denotes the top-*p* genes sifted by the feature selection procedures.

[b]genes denote the number of genes passed DA screening across the training and test domains for each drug.

[c]The bold-faced values indicate the highest CV score among the three predictors for a drug.

Table S1 (continued)

Method / Drug (test set)	**LogitDA_0.8**			**LogitDA_0.9**		
	p / genes	λ	CV score	*p* / genes	λ	CV score
Docetaxel (GSE6434) n=24	170	0.242	0.75	50	0.424	0.66
	223			59		
Erlotinib (GSE30072) n=25	100	0.962	0.78	50	1.122	0.68
	391			90		
Sorafenib (GSE30072) n=37	90	1.122	0.67	50	1.122	0.63
	752			207		
Cetuximab (PDX) n=60	150	0.041	0.84	50	0.048	0.80
	440			157		
Erlotinib (PDX) n=21	50	0.521	0.82	50	1.122	0.79
	499			163		
Gemcitabine (PDX) n=25	70	0.521	0.69	50	0.242	0.67
	541			151		
Paclitaxel (PDX) n=43	50	1.122	0.64	50	1.039	0.49
	311			78		
Cisplatin (TCGA) n=66	110	0.192	0.67	50	1.066	0.60
	293			79		
Docetaxel (TCGA) n=16	240	0.424	0.77	80	0.236	0.74
	691			193		

Gemcitabine (TCGA) n=57	70	0.192	0.72	50	0.414	0.66
	399			94		

Table S2A. The sample information of the source and target domains for the ten drugs.

	Training set				Test set				
**Drug (test data)**	Samples	NS[a]	NR[a]	NS/NR	Samples	NS	NR	NS/NR	r
**Docetaxel (GSE6434)**	850	564	286	1.97	24	10	14	0.71	2.77
**Erlotinib (GSE30072)**	370	28	342	0.08	25	11	14	0.79	0.1
**Sorafenib (GSE30072)**	403	117	286	0.41	37	21	16	1.31	0.31
**Cetuximab (PDX)**	877	40	837	0.05	60	5	55	0.09	0.56
**Erlotinib (PDX)**	370	28	342	0.08	21	3	18	0.17	0.47
**Gemcitabine (PDX)**	866	680	186	3.66	25	7	18	0.39	9.38
**Paclitaxel (PDX)**	399	284	115	2.47	43	5	38	0.13	19
**Cisplatin (TCGA)**	850	275	575	0.48	66	60	6	10.00	0.05
**Docetaxel (TCGA)**	850	564	286	1.97	16	8	8	1.00	1.97
**Gemcitabine (TCGA)**	866	680	186	3.66	57	21	36	0.58	6.31

Note: NS and NR denote the number of sensitive and resistant cell lines, and r denotes the ratio of NS/NR in the source versus the target domain.

Table S2B. The predicted results of LogitDA and the true values for Erlotinib with the adjusted cutoff

Erlotinib (GSE33072), LogitDA$_{_0.7,}$ using the adjusted cutoff 0.09					
clinical_name	*EGFR*	*KRAS*	yhat	predicted y	y
GSM677318	-1.27003	0.5921	0.108	1	1
GSM677321	-0.44741	0.9705	0.083	0	1
GSM677326	-0.32983	-0.3995	0.113	1	1
GSM677327	0.363664	1.8198	0.072	0	0
GSM789976	1.734497	-0.129	0.063	0	0
GSM789977	-0.21588	1.0234	0.069	0	0
GSM789980	-1.36227	-2.4861	0.039	0	0
GSM789982	-0.0265	0.3341	0.085	0	1
GSM789984	0.202044	0.6343	0.108	1	1
GSM789985	-0.86409	-0.0179	0.086	0	0
GSM789994	0.261084	-1.2116	0.056	0	0
GSM789999	0.297998	-0.4441	0.133	1	1
GSM790000	-0.14663	0.4081	0.062	0	0
GSM790001	-1.48974	1.1048	0.087	0	1
GSM790008	1.854998	-1.0632	0.064	0	0
GSM790017	0.022626	-1.1713	0.051	0	0
GSM790022	0.212112	0.1203	0.09	1	1
GSM790023	1.154709	-0.0062	0.067	0	0
GSM790028	1.396441	0.6781	0.082	0	1
GSM790032	0.109333	-0.4844	0.031	0	0
GSM790033	1.61096	0.9019	0.073	0	1
GSM790034	-0.15515	0.1444	0.067	0	1
GSM790041	-0.82795	1.1084	0.072	0	0
GSM790042	-0.186	-1.4189	0.039	0	0
GSM790046	-1.89901	-1.0081	0.051	0	0

Note: Those marked in yellow are *KRAS* mutant patients with NSCLC. Yhat and predicted y denote the predicted and associated dichotomized values of LogitDA.

Table S3. Comparison of LogitDA/KNNDA with those without DA in terms of prediction AUC for the ten drugs

Method / Drug	KNNDA-DA			KNNDA			LogitDA-DA		LogitDA	
	p	K	AUC	p	K	AUC	p	AUC	p	AUC
Docetaxel (GSE6434) n=24	140	23	0.65	110	23	0.87	400	0.76	170	0.76
Erlotinib (GSE33072) n=25	100	19	0.56	220	9	0.90	400	0.70	50	0.94
Sorafenib (GSE33072) n=37	50	9	0.50	120	17	0.71	500	0.45	110	0.70
Cetuximab (PDX) n=60	700	23	0.40	110	23	0.95	400	0.60	130	0.93
Erlotinib (PDX) n=21	300	17	0.81	60	19	1.00	400	0.30	50	1.00
Gemcitabine (PDX) n=25	340	21	0.48	240	17	0.62	300	0.56	100	0.83
Paclitaxel (PDX) n=43	80	7	0.51	100	9	0.65	400	0.63	50	0.68
Cisplatin (TCGA) n=66	280	19	0.58	190	29	0.67	400	0.38	110	0.62
Docetaxel (TCGA) n=16	160	23	0.63	200	25	0.77	400	0.48	650	0.81
Gemcitabine (TCGA) n=57	340	21	0.53	180	29	0.68	300	0.48	140	0.62

Table S4. The uncovered pathways of the fitted genes of LogitDA and KNNDA for Erlotinib (GSE33072)

Ingenuity Canonical Pathways	*p*-value	Molecules	References
**Metabolic process**			
Purine Nucleotides De Novo Biosynthesis II	0.004	ADSL, PFAS	1,2
Pregnenolone Biosynthesis	0.010	CYP46A1, MICAL3	3,4
Histidine Degradation VI	0.012	CYP46A1, MICAL3	3,4
Ubiquinol-10 Biosynthesis (Eukaryotic)	0.014	CYP46A1, MICAL3	3,4
UDP-D-xylose and UDP-D-glucuronate Biosynthesis	0.018	UXS1	-*
Inosine-5'-phosphate Biosynthesis II	0.028	ADSL	1
Uridine-5'-phosphate Biosynthesis	0.028	CAD	5
Diphthamide Biosynthesis	0.028	EEF2	6
5-aminoimidazole Ribonucleotide Biosynthesis I	0.028	PFAS	2
Thiosulfate Disproportionation III (Rhodanese)	0.028	MOCS3	7
Branched-chain α-keto acid Dehydrogenase Complex	0.036	BCKDHB	8
Molybdenum Cofactor Biosynthesis	0.036	MOCS3	7
**Epigenetic regulation**			
DNA Methylation and Transcriptional Repression Signaling	0.006	H4C13, MTA3 RBBP7	9,10
**DNA repair**			
NER (Nucleotide Excision Repair, Enhanced Pathway)	0.014	CHAF1A, H4C13, LIG1, UVSSA	11-14

*The notation - denotes "not-related to cancer".

References:

1. Taha-Mehlitz S, Bianco G, Coto-Llerena M, Kancherla V, Bantug GR, Gallon J, Ercan C, Panebianco F, Eppenberger-Castori S, von Strauss M, Staubli S, Bolli M, Peterli R, Matter MS,

Terracciano LM, von Flüe M, Ng CKY, Soysal SD, Kollmar O, Piscuoglio S. Adenylosuccinate lyase is oncogenic in colorectal cancer by causing mitochondrial dysfunction and independent activation of NRF2 and mTOR-MYC-axis. Theranostics. 2021 Feb 15;11(9):4011-4029.

2. Ali ES, Sahu U, Villa E, O'Hara BP, Gao P, Beaudet C, Wood AW, Asara JM, Ben-Sahra I. ERK2 Phosphorylates PFAS to Mediate Posttranslational Control of De Novo Purine Synthesis. Mol Cell. 2020 Jun 18;78(6):1178-1191.e6.
3. Han M, Wang S, Yang N, Wang X, Zhao W, Saed HS, Daubon T, Huang B, Chen A, Li G, Miletic H, Thorsen F, Bjerkvig R, Li X, Wang J. Therapeutic implications of altered cholesterol homeostasis mediated by loss of CYP46A1 in human glioblastoma. EMBO Mol Med. 2020 Jan 9;12(1):e10924.
4. Tominaga K, Minato H, Murayama T, Sasahara A, Nishimura T, Kiyokawa E, Kanauchi H, Shimizu S, Sato A, Nishioka K, Tsuji EI, Yano M, Ogawa T, Ishii H, Mori M, Akashi K, Okamoto K, Tanabe M, Tada KI, Tojo A, Gotoh N. Semaphorin signaling via MICAL3 induces symmetric cell division to expand breast cancer stem-like cells. Proc Natl Acad Sci U S A. 2019 Jan 8;116(2):625-630.
5. Tu HF, Ko CJ, Lee CT, Lee CF, Lan SW, Lin HH, Lin HY, Ku CC, Lee DY, Chen IC, Chuang YH, Del Caño-Ochoa F, Ramón-Maiques S, Ho CC, Lee MS, Chang GD. Afatinib Exerts Immunomodulatory Effects by Targeting the Pyrimidine Biosynthesis Enzyme CAD. Cancer Res. 2021 Jun 15;81(12):3270-3282.
6. McDermott MS, Browne BC, Conlon NT, O'Brien NA, Slamon DJ, Henry M, Meleady P, Clynes M, Dowling P, Crown J, O'Donovan N. PP2A inhibition overcomes acquired resistance to HER2 targeted therapy. Mol Cancer. 2014 Jun 24;13:157.
7. Guerrero Llobet S, Bhattacharya A, Everts M, Kok K, van der Vegt B, Fehrmann RSN, van Vugt MATM. An mRNA expression-based signature for oncogene-induced replication-stress. Oncogene. 2022 Feb;41(8):1216-1224.
8. Zhang S, Zeng X, Lin S, Liang M, Huang H. Identification of seven-gene marker to predict the survival of patients with lung adenocarcinoma using integrated multi-omics data analysis. J Clin Lab Anal. 2022 Feb;36(2):e24190.
9. Du L, Wang L, Gan J, Yao Z, Lin W, Li J, Guo Y, Chen Y, Zhou F, Jim Yeung SC, Coppes RP, Zhang D, Zhang H. MTA3 Represses Cancer Stemness by Targeting the SOX2OT/SOX2 Axis. iScience. 2019 Dec 20;22:353-368.
10. Yu N, Zhang P, Wang L, He X, Yang S, Lu H. RBBP7 is a prognostic biomarker in patients with esophageal squamous cell carcinoma. Oncol Lett. 2018 Dec;16(6):7204-7211.
11. Dong S, Li W, Wang L, Hu J, Song Y, Zhang B, Ren X, Ji S, Li J, Xu P, Liang Y, Chen G, Lou JT, Yu W. Histone-Related Genes Are Hypermethylated in Lung Cancer and Hypermethylated *HIST1H4F* Could Serve as a Pan-Cancer Biomarker. Cancer Res. 2019 Dec 15;79(24):6101-6112.
12. Wang T, Chen X, Jing F, Li Z, Tan H, Luo Y, Shi H. Identifying the hub genes in non-small cell lung cancer by integrated bioinformatics methods and analyzing the prognostic values. Pathol Res Pract. 2021 Dec;228:153654.
13. Ali R, Alabdullah M, Algethami M, Alblihy A, Miligy I, Shoqafi A, Mesquita KA, Abdel-Fatah T, Chan SY, Chiang PW, Mongan NP, Rakha EA, Tomkinson AE, Madhusudan S. Ligase 1 is a predictor of platinum resistance and its blockade is synthetically lethal in XRCC1 deficient epithelial ovarian cancers. Theranostics. 2021 Jul 25;11(17):8350-8361.
14. Sato M, Liebau AW, Liu Z, Liu L, Rabadan R, Gautier J. The UVSSA complex alleviates MYC-driven transcription stress. J Cell Biol. 2021 Feb 1;220(2):e201807163.

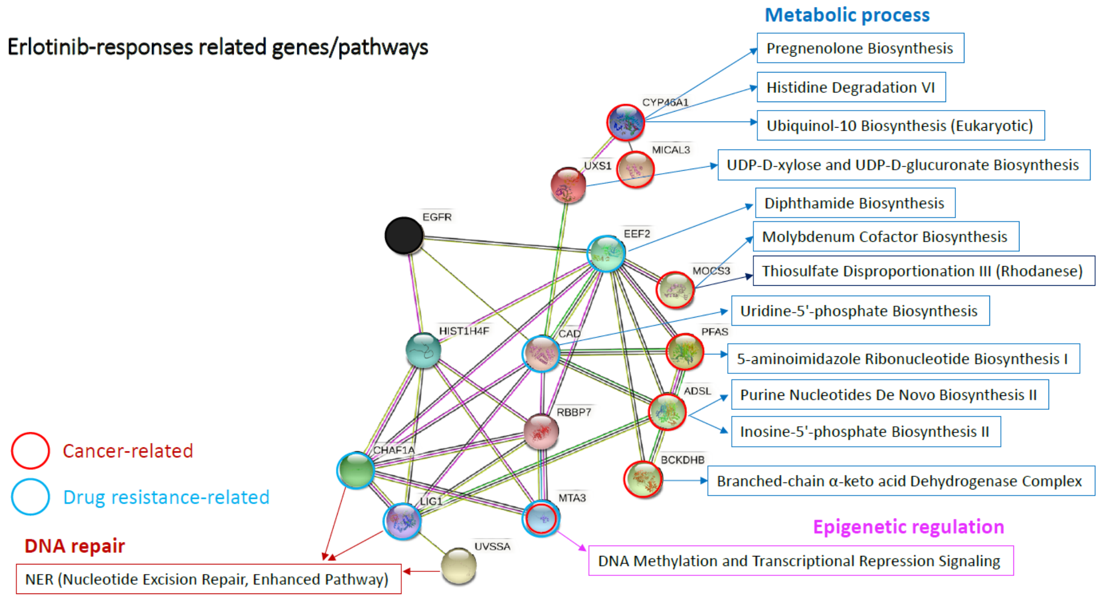
Erlotinib-responses related genes/pathways
Metabolic process
Pregnenolone Biosynthesis
Histidine Degradation VI
Ubiquinol-10 Biosynthesis (Eukaryotic)
UDP-D-xylose and UDP-D-glucuronate Biosynthesis
Diphthamide Biosynthesis
Molybdenum Cofactor Biosynthesis
Thiosulfate Disproportionation III (Rhodanese)
Uridine-5'-phosphate Biosynthesis
5-aminoimidazole Ribonucleotide Biosynthesis I
Purine Nucleotides De Novo Biosynthesis II
Inosine-5'-phosphate Biosynthesis II
Branched-chain α-keto acid Dehydrogenase Complex
Epigenetic regulation
DNA Methylation and Transcriptional Repression Signaling
DNA repair
NER (Nucleotide Excision Repair, Enhanced Pathway)
Cancer-related
Drug resistance-related
CYP46A1
MICAL3
UXS1
EGFR
EEF2
MOCS3
HIST1H4F
CAD
PFAS
ADSL
RBBP7
CHAF1A
BCKDHB
LIG1
MTA3
UVSSA

Table S5. The uncovered pathways of the fitted genes of LogitDA for Cetuximab (PDX) with prediction AUC= 0.93

Ingenuity Canonical Pathways	p-value	Molecules	References
**DNA repair**			
Nucleotide Excision Repair Pathway	0.019055	CDK7, POLR2B	1-4
NER (Nucleotide Excision Repair, Enhanced Pathway)	0.024547	CDK7, POLR2B, TCEA1	1-5
BER (Base Excision Repair) Pathway	0.029512	OGG1, TDG	6-8
UVB-Induced MAPK Signaling	0.039811	EIF4EBP1, PIK3C2A	9,10
Role of p14/p19ARF in Tumor Suppression	0.012589	PIK3C2A, POLR3D	10,11
**Metabolic pathways**			
NAD Signaling Pathway	0.012023	ACADM, NADK, PIK3C2A, POLR2B	4, 10, 12,13
Assembly of RNA Polymerase II Complex	0.003548	CDK7, POLR2B, TAF9	1-4, 14
Putrescine Biosynthesis III	0.012303	ODC1	15
Tetrahydrobiopterin Biosynthesis I/II	0.018197	SPR	16
Pentose Phosphate Pathway (Non-oxidative Branch)	0.036308	TALDO1	17
GDP-mannose Biosynthesis	0.036308	PMM1	-*
**Lysosome-associated, e.g., autophagy**			
Iron homeostasis signaling pathway	0.047863	ACO2, ATP6V1B2, MCOLN1	18-20
CLEAR Signaling Pathway	0.028840	ATP6V1B2, IGF1R, LAMP1, MCOLN1, UVRAG	18-24
**Others**			
Glioma Signaling	0.038905	CDKN2C, IGF1R, PIK3C2A	10, 21, 22, 25,
EIF2 Signaling	0.040738	EIF2B3, IGF1R, PIK3C2A, RPL38	10, 21,22, 26,27

*The notation - denotes "not-related to cancer".

References:

1. Jagomast T, Idel C, Klapper L, Kuppler P, Offermann A, Dreyer E, Bruchhage KL, Ribbat-Idel J, Perner S. CDK7 Predicts Worse Outcome in Head and Neck Squamous-Cell Cancer. Cancers (Basel). 2022 Jan 19;14(3):492.
2. Rusan M, Li K, Li Y, Christensen CL, Abraham BJ, Kwiatkowski N, Buczkowski KA, Bockorny B, Chen T, Li S, Rhee K, Zhang H, Chen W, Terai H, Tavares T, Leggett AL, Li T, Wang Y, Zhang T, Kim TJ, Hong SH, Poudel-Neupane N, Silkes M, Mudianto T, Tan L, Shimamura T, Meyerson M, Bass AJ, Watanabe H, Gray NS, Young RA, Wong KK, Hammerman PS. Suppression of Adaptive Responses to Targeted Cancer Therapy by Transcriptional Repression. Cancer Discov. 2018 Jan;8(1):59-73.

3. Terai H, Kitajima S, Potter DS, Matsui Y, Quiceno LG, Chen T, Kim TJ, Rusan M, Thai TC, Piccioni F, Donovan KA, Kwiatkowski N, Hinohara K, Wei G, Gray NS, Fischer ES, Wong KK, Shimamura T, Letai A, Hammerman PS, Barbie DA. ER Stress Signaling Promotes the Survival of Cancer "Persister Cells" Tolerant to EGFR Tyrosine Kinase Inhibitors. Cancer Res. 2018 Feb 15;78(4):1044-1057.
4. Michiels S, Danoy P, Dessen P, Bera A, Boulet T, Bouchardy C, Lathrop M, Sarasin A, Benhamou S. Polymorphism discovery in 62 DNA repair genes and haplotype associations with risks for lung and head and neck cancers. Carcinogenesis. 2007 Aug;28(8):1731-9.
5. You S, Wang F, Hu Q, Li P, Zhang C, Yu Y, Zhang Y, Li Q, Bao Q, Liu P, Li J. Abnormal expression of YEATS4 associates with poor prognosis and promotes cell proliferation of hepatic carcinoma cell by regulation the TCEA1/DDX3 axis. Am J Cancer Res. 2018 Oct 1;8(10):2076-2087.
6. Vlahopoulos S, Adamaki M, Khoury N, Zoumpourlis V, Boldogh I. Roles of DNA repair enzyme OGG1 in innate immunity and its significance for lung cancer. Pharmacol Ther. 2019 Feb;194:59-72.
7. Dziaman T, Banaszkiewicz Z, Roszkowski K, Gackowski D, Wisniewska E, Rozalski R, Foksinski M, Siomek A, Speina E, Winczura A, Marszalek A, Tudek B, Olinski R. 8-Oxo-7,8-dihydroguanine and uric acid as efficient predictors of survival in colon cancer patients. Int J Cancer. 2014 Jan 15;134(2):376-83.
8. Zhu Y, Li J, Bo H, He D, Xiao M, Xiang L, Gong L, Hu Y, Zhang Y, Cheng Y, Deng L, Zhu R, Ma Y, Cao K. LINC00467 is up-regulated by TDG-mediated acetylation in non-small cell lung cancer and promotes tumor progression. Oncogene. 2020 Sep;39(38):6071-6084.
9. Tang Y, Luo J, Yang Y, Liu S, Zheng H, Zhan Y, Fan S, Wen Q. Overexpression of p-4EBP1 associates with p-eIF4E and predicts poor prognosis for non-small cell lung cancer patients with resection. PLoS One. 2022 Jun 23;17(6):e0265465.
10. Sullivan I, Salazar J, Arqueros C, Andrés M, Sebio A, Majem M, Szafranska J, Martínez E, Páez D, López-Pousa A, Baiget M, Barnadas A. KRAS genetic variant as a prognostic factor for recurrence in resectable non-small cell lung cancer. Clin Transl Oncol. 2017 Jul;19(7):884-890.
11. Hao Z, Wang S, Zheng Z, Li J, Fu W, Han D, Huang Y, Lin Q, Xian S, Yan P, Li M, Lin R, Meng T, Zhang J, Huang Z. Prognostic Bone Metastasis-Associated Immune-Related Genes Regulated by Transcription Factors in Mesothelioma. Biomed Res Int. 2022 Jan 27;2022:9940566. doi: 10.11 55/2022/9940566. eCollection 2022.
12. Ma APY, Yeung CLS, Tey SK, Mao X, Wong SWK, Ng TH, Ko FCF, Kwong EML, Tang AHN, Ng IO, Cai SH, Yun JP, Yam JWP. Suppression of ACADM-Mediated Fatty Acid Oxidation Promotes Hepatocellular Carcinoma via Aberrant CAV1/SREBP1 Signaling. Cancer Res. 2021 Jul 1;81(13):3679-3692.
13. Schild T, McReynolds MR, Shea C, Low V, Schaffer BE, Asara JM, Piskounova E, Dephoure N, Rabinowitz JD, Gomes AP, Blenis J. NADK is activated by oncogenic signaling to sustain pancreatic ductal adenocarcinoma. Cell Rep. 2021 Jun 15;35(11):109238.
14. Yoon JW, Lamm M, Iannaccone S, Higashiyama N, Leong KF, Iannaccone P, Walterhouse D. p53 modulates the activity of the GLI1 oncogene through interactions with the shared coactivator TAF9. DNA Repair (Amst). 2015 Oct;34:9-17.
15. Lam SK, U KP, Li YY, Xu S, Cheng PN, Ho JC. Inhibition of ornithine decarboxylase 1 facilitates pegylated arginase treatment in lung adenocarcinoma xenograft models. Oncol Rep. 2018 Oct;40(4):1994-2004.

16. Wu Y, Du H, Zhan M, Wang H, Chen P, Du D, Liu X, Huang X, Ma P, Peng D, Sun L, Yuan S, Ding J, Lu L, Jiang J. Sepiapterin reductase promotes hepatocellular carcinoma progression via FoxO3a/Bim signaling in a nonenzymatic manner. Cell Death Dis. 2020 Apr 20;11(4):248.
17. Grammatikopoulos T, Hadzic N, Foskett P, Strautnieks S, Samyn M, Vara R, Dhawan A, Hertecant J, Al Jasmi F, Rahman O; University of Washington Center for Mendelian Genomics, Deheragoda M, Bull LN, Thompson RJ. Liver Disease and Risk of Hepatocellular Carcinoma in Children With Mutations in TALDO1. Hepatol Commun. 2022 Mar;6(3):473-479.
18. Pastvova N, Havlasek J, Dolezel P, Kikalova K, Studentova H, Zemankova A, Melichar B, Mlejnek P. Changes in expression of lysosomal membrane proteins in leucocytes of cancer patients treated with tyrosine kinase inhibitors. Cancer Chemother Pharmacol. 2021 Jul;88(1):89-98.
19. Xing Y, Wei X, Liu Y, Wang MM, Sui Z, Wang X, Zhu W, Wu M, Lu C, Fei YH, Jiang Y, Zhang Y, Wang Y, Guo F, Cao JL, Qi J, Wang W. Autophagy inhibition mediated by MCOLN1/TRPML1 suppresses cancer metastasis via regulating a ROS-driven TP53/p53 pathway. Autophagy. 2022 Aug;18(8):1932-1954.
20. Yin C, Zhang H, Liu X, Zhang H, Zhang Y, Bai X, Wang L, Li H, Li X, Zhang S, Zhang L, Zhang Y. Downregulated MCOLN1 Attenuates The Progression Of Non-Small-Cell Lung Cancer By Inhibiting Lysosome-Autophagy. Cancer Manag Res. 2019 Sep 23;11:8607-8617.
21. Suda K, Mizuuchi H, Sato K, Takemoto T, Iwasaki T, Mitsudomi T. The insulin-like growth factor 1 receptor causes acquired resistance to erlotinib in lung cancer cells with the wild-type epidermal growth factor receptor. Int J Cancer. 2014 Aug 15;135(4):1002-6.
22. Li X, Xu L, Li H, Zhao L, Luo Y, Zhu Z, Liu Y, Qu X. Cetuximab-induced insulin-like growth factor receptor I activation mediates cetuximab resistance in gastric cancer cells. Mol Med Rep. 2015 Jun;11(6):4547-54.
23. Wang Q, Yao J, Jin Q, Wang X, Zhu H, Huang F, Wang W, Qiang J, Ni Q. LAMP1 expression is associated with poor prognosis in breast cancer. Oncol Lett. 2017 Oct;14(4):4729-4735.
24. Feng X, Jia Y, Zhang Y, Ma F, Zhu Y, Hong X, Zhou Q, He R, Zhang H, Jin J, Piao D, Huang H, Li Q, Qiu X, Zhang Z. Ubiquitination of UVRAG by SMURF1 promotes autophagosome maturation and inhibits hepatocellular carcinoma growth. Autophagy. 2019 Jul;15(7):1130-1149.
25. Li GS, Chen G, Liu J, Tang D, Zheng JH, Luo J, Jin MH, Lu HS, Bao CX, Tian J, Deng WS, Fu JW, Feng Y, Zeng NY, Zhou HF, Kong JL. Clinical significance of cyclin-dependent kinase inhibitor 2C expression in cancers: from small cell lung carcinoma to pan-cancers. BMC Pulm Med. 2022 Jun 24;22(1):246.
26. Ye Q, Falatovich B, Singh S, Ivanov AV, Eubank TD, Guo NL. A Multi-Omics Network of a Seven-Gene Prognostic Signature for Non-Small Cell Lung Cancer. Int J Mol Sci. 2021 Dec 25;23(1):219.
27. López-Cortés A, Cabrera-Andrade A, Vázquez-Naya JM, Pazos A, Gonzáles-Díaz H, Paz-Y-Miño C, Guerrero S, Pérez-Castillo Y, Tejera E, Munteanu CR. Prediction of breast cancer proteins involved in immunotherapy, metastasis, and RNA-binding using molecular descriptors and artificial neural networks. Sci Rep. 2020 May 22;10(1):8515.

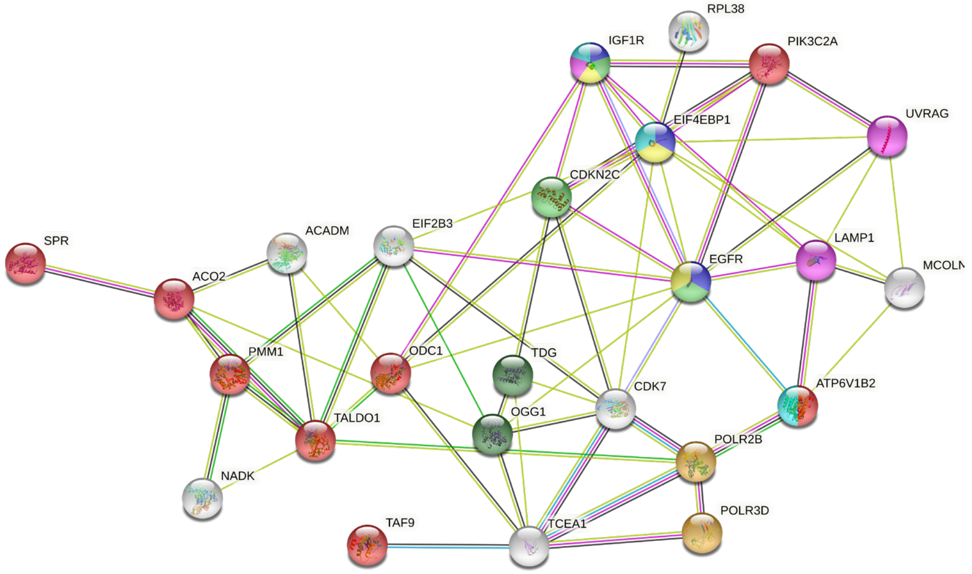

KEGG Pathways

pathway	description	count in network	strength	false discovery rate
hsa01100	Metabolic pathways	8 of 1447	0.64	0.0493
hsa01521	EGFR tyrosine kinase inhibitor resistance	3 of 78	1.48	0.0493
hsa01522	Endocrine resistance	3 of 95	1.39	0.0493
hsa04066	HIF-1 signaling pathway	3 of 106	1.35	0.0493
hsa04140	Autophagy - animal	3 of 130	1.26	0.0493
hsa04150	mTOR signaling pathway	3 of 151	1.19	0.0493
hsa03020	RNA polymerase	2 of 31	1.7	0.0493
hsa03410	Base excision repair	2 of 33	1.68	0.0493

Table S6. The test AUC of KNNDA_0.7 with various values of hyperparameter K

Drug	p[a] / genes[b]											
Docetaxel (GSE6434) n=24	110	K	13	15	17	19	21	**23**[c]	25	27	29	31
	437	Test AUC	0.82	0.82	0.84	0.86	0.84	**0.87**	0.86	0.90	0.89	0.81
Erlotinib (GSE30072) n=25	220	K	3	5	7	**9**	11	13	15	17	19	21
	860	Test AUC	0.85	0.94	0.92	**0.90**	0.93	0.92	0.92	0.92	0.91	0.90
Sorafenib (GSE30072) n=37	120	K	3	5	7	9	11	13	15	**17**	19	21
	1000	Test AUC	0.60	0.63	0.64	0.67	0.65	0.63	0.70	**0.71**	0.69	0.68
Cetuximab (PDX) n=60	110	K	13	15	17	19	21	**23**	25	27	29	31
	827	Test AUC	0.94	0.95	0.95	0.96	0.96	**0.95**	0.94	0.95	0.96	0.96
Erlotinib (PDX) n=21	60	K	3	5	7	9	11	13	15	17	**19**	21
	877	Test AUC	0.89	0.91	1	0.96	0.98	1	0.97	0.99	**1**	0.99
Gemcitabine (PDX) n=21	240	K	13	15	**17**	19	21	23	25	27	29	31
	1000	Test AUC	0.64	0.66	**0.62**	0.63	0.62	0.64	0.65	0.66	0.64	0.66
Paclitaxel (PDX) n=43	100	K	3	5	7	**9**	11	13	15	17	19	21
	643	Test AUC	0.47	0.59	0.59	**0.65**	0.64	0.69	0.67	0.70	0.67	0.67
Cisplatin (TCGA) n=66	190	K	13	15	17	19	21	23	25	27	**29**	31
	628	Test AUC	0.78	0.76	0.74	0.72	0.71	0.70	0.68	0.66	**0.67**	0.67
Docetaxel (TCGA) n=16	200	K	13	15	17	19	21	23	**25**	27	29	31
	1000	Test AUC	0.87	0.86	0.87	0.86	0.83	0.77	**0.77**	0.78	0.78	0.77
Gemcitabine (TCGA) n=57	180	K	13	15	17	19	21	23	25	27	**29**	31
	841	Test AUC	0.67	0.70	0.71	0.70	0.68	0.63	0.65	0.67	**0.68**	0.68

[a]*p* denotes the top-*p* genes sifted by the feature selection procedures.
[b]genes denotes the number of genes passed DA screening across the training and test domains for each drug.
[c]The bold-faced value K denotes the 5-fold CV determined K of KNN.
